# A Screening Test for *HLA-B^∗^15:02* in a Large United States Patient Cohort Identifies Broader Risk of Carbamazepine-Induced Adverse Events

**DOI:** 10.3389/fphar.2019.00149

**Published:** 2019-03-26

**Authors:** Hua Fang, Xiequn Xu, Kulvinder Kaur, Matthew Dedek, Guang-dan Zhu, Bae J. Riley, Frank G. Espin, Andria L. Del Tredici, Tanya A. Moreno

**Affiliations:** Millennium Health, San Diego, CA, United States

**Keywords:** *HLA-B^∗^15:02*, pharmacogenetics, carbamazepine, SJS, tagging SNP, population screening

## Abstract

**Purpose:** *HLA-B^∗^15:02* is strongly associated with life-threatening severe skin hypersensitivity reactions in patients treated with carbamazepine (CBZ) and structurally related medications. FDA-approved labeling recommends *HLA-B^∗^15:02* screening before CBZ therapy in patients of Asian ancestry. In this study, we aimed to (a) identify a direct method for screening *HLA-B^∗^15:02*, and (b) evaluate prevalence in a large cohort of United States patients.

**Methods:** Candidate genetic markers were identified by mining public data. Association was tested in 28,897 individuals by comparing SNP results with high-resolution HLA typing. Retrospective analysis of de-identified SNP and ethnicity data from 130,460 individuals was performed to evaluate the ethnic distribution of *HLA-B^∗^15:02* in the United States.

**Results:** 28,897 United States individuals showed 100% concordance between *HLA-B^∗^15:02* and the minor allele of rs144012689 (100% sensitivity/99.97% specificity). Retrospective analysis of 160 positive individuals (66 with physician-reported ethnicity) notably included 28 Asians (42%), 15 African Americans (22%), 11 Caucasians (17%), 2 Hispanics (3%), and 10 “Other” (15%).

**Conclusion:** Screening United States patients for *HLA-B^∗^15:02* without ethnicity-based preselection identifies more than twice the number of carriers at risk of CBZ-related adverse events than screening patients of Asian ancestry alone. Risk assessment based on ethnicity assumptions may not identify a large portion of at-risk patients in the ethnically diverse United States population.

## Introduction

The human leukocyte antigen B gene (*HLA-B*), located on the short arm of chromosome 6 (6p21.3), is a member of the human major histocompatibility complex (MHC) class I complex that encodes a cell surface protein involved in presenting antigens to the immune system (Leckband et al., 2013). HLA proteins present a wide variety of peptides for immune recognition, and the *HLA* genes are among the most polymorphic in the human genome. According to the World Health Organization Nomenclature Committee for Factors of the HLA System^1^, there are nearly 4,000 identified *HLA-B* alleles. Among these, some are associated with drug-induced adverse responses. One such allele, *HLA-B*^∗^*15:02*, is strongly associated with an increased risk of severe and sometimes life-threatening inflammatory adverse reactions in skin and mucous membranes, known as Stevens-Johnson syndrome (SJS) and toxic epidermal necrolysis (TEN), in patients taking carbamazepine (CBZ) (Chung et al., 2004; Chen et al., 2011; Yip et al., 2012) and CBZ-related drugs (e.g., phenytoin, lamotrigine) (Man et al., 2007; Bloch et al., 2014). The United States Food and Drug Administration (FDA) recommends screening for *HLA-B^∗^15:02* allele before CBZ therapy in patients of Asian ancestry, who are known to have high frequencies of the *HLA-B^∗^15:02* allele (FDA, 2007, 2015).

Detection of *HLA-B^∗^15:02* is feasible but technically challenging due to the highly polymorphic nature of various *HLA-B* alleles. Among many, there are four major approaches currently available: (1) direct sequencing by Sanger or next-generation sequencing (NGS); (2) sequence-specific oligonucleotide probe hybridization (SSOP) (Fleischhauer et al., 1995; Cao et al., 1999); (3) sequence-specific PCR (SSP-PCR) (Bunce et al., 1995); and (4) tag-SNP methods (SNP, single nucleotide polymporphism). These methods may be either highly accurate but fairly difficult to access due to complex processing and the requirement of expensive equipment, or less expensive and easier to perform but only appropriate for low-throughput testing. Direct sequencing is the most accurate but it can be time-consuming and expensive, and it requires special expertise to analyze the data. A significant challenge for *HLA-B* testing is the issue of low specificity due to genomic complexity and polymorphism. In particular, a two-SNP haplotype consisting of the minor alleles of rs2844682 and rs3909184 as proxy tagging SNPs for *HLA-B^∗^15:02* allele (de Bakker et al., 2006) has been shown to provide very low clinical accuracy in recent studies (He et al., 2015; Zhu et al., 2015).

Here, we report that a SNP within the *HLA-B* gene (rs144012689) is strongly associated with *HLA-B^∗^15:02*. An important outcome from this study was the unexpected finding that a significant number of *HLA-B^∗^15:02* carriers were reported to have non-Asian ethnicity, indicating that in the United States, Asian ethnicity may not be an appropriate limitation for *HLA-B^∗^15:02* testing.

## Materials and Methods

### Biobank Genomic DNA Samples and Study Samples

A set of 32 genomic DNA (gDNA) samples known to contain *HLA-B^∗^15:02* (positive reference samples) were purchased from Coriell Cell Repositories (Camden, NJ, United States) and the International Histocompatibility Working Group (IHWG) Cell and DNA bank (Seattle, WA, United States). The samples used were: NA17019, NA23093, NA23090, NA18547, HG00406, HG00536, HG00560, HG00595, HG00620, HG00671, HG00701, NA18559, NA18639, NA18630, NA18152, NA18674, NA17999, NA18127, NA17988, NA17997, NA18118, NA18122, NA17982, NA17977, NA17969, IHW09182, IHW09185, IHW09186, IHW09189, IHW09199, IHW09237, and IHW09432. In addition, de-identified data from samples of 130,460 patients undergoing clinical pharmacogenetics testing were included in this study. The study was conducted under a protocol reviewed and approved by the Institutional Review Board at Aspire IRB, Santee, CA, United States.

### *HLA-B* Genomic Sequence Alignment Analysis

The genomic sequences of 344 non-*B^∗^15:02 HLA-B* references and 9 *HLA-B^∗^15:02* subtypes (*HLA-B^∗^15:02:01* – *HLA-B^∗^15:02:09*) from the World Health Organization Nomenclature Committee for Factors of the HLA system^2^ (Robinson et al., 2000, 2015) were aligned and compared. In brief, each position of the alignment was applied to a counting method to check how many non-*HLA-B^∗^15:02* alleles shared any given SNP with *HLA-B^∗^15:02*; a window containing the least shared locations was chosen.

### 1000 Genomes Data

Population genetics data and the individual genotype data on rs144012689 were extracted from The 1000 Genomes (International HapMap Consortium, 2003) Phase 3 database^3^.

### *HLA-B* Genotypes of the 1000 Genomes Samples on NCBI dbMHC Portal

*HLA-B* genotype results of the 1000 Genomes samples were generated in a prior research study (Gourraud et al., 2014). Data were accessed at^4^.

### NGS-Based *HLA-B* Typing and Genotyping of rs144012689

*HLA-B* typing was performed by Histogenetics LLC (Ossining, NY, United States). Each sample was sequenced by high resolution *HLA-B* Gold typing using NGS for exons 2/3 and intron 5 for rs144012689.

### PCR-Based Genotyping for rs144012689

An assay was designed for rs144012689 using standard primer and probe design techniques, also taking into account the complexity of the *HLA-B* locus surrounding this SNP (Fang et al., 2015). The probe detects “T” as minor allele on the minus strand or “A” as the major allele. An adjacent SNP, rs2596496 (“G” as minor allele on the minus strand, minor allele frequency = 0.25), was taken into consideration during probe design. Probes were purchased from Life Technologies (Foster City, CA, United States). Primers were designed at the conserved regions surrounding rs144012689 and purchased from Integrated DNA Technologies (Coralville, IA, United States; Fang et al., 2015). TaqMan-based PCR genotyping for rs144012689 was carried out on the Fluidigm Biomark HD. A representative genotype scatter plot is shown in Supplementary Figure S1. gDNA was extracted from oral swabs (OCD-100, DNA Genotek, Ottawa, ON, Canada) using Chemagic DNA Saliva Kit (Perkin Elmer, Waltham, MA, United States) and tested using the standard Fluidigm Biomark PCR protocol on GE96.96 dynamic arrays. Patent granted by the United States Patent and Trademark Office (US9932638B2).

### Data Analysis and Statistics

Sequencing data analysis was performed by Histogenetics LLC, Ossining, NY, United States. SNP genotyping analysis software (Fluidigm) was used to analyze the results from PCR assays. GraphPad software was used to perform the Chi-square test. Microsoft Excel was used to assemble data tables and charts.

### Data Availability

All relevant data generated and analyzed in this study are included within the manuscript and/or Supplementary Files, or accessible in the referenced publicly accessible repositories.

## Results

### rs144012689 as a Potential Genetic Marker for *HLA-B^∗^15:02*

In order to identify *HLA-B^∗^15:02*-specific regions within the *HLA-B* gene to distinguish it from other highly homologous *HLA-B* alleles, we examined the sequences of 9 *HLA-B^∗^15:02* subtype references (*HLA-B^∗^15:02:01* – *B^∗^15:02:09*) as well as 344 available non-*B^∗^15:02* *HLA-B* reference sequences. Within the entirety of *HLA-B* genomic sequence, there was no single SNP that was unique to *HLA-B^∗^15:02* and its subtypes. However, one SNP in intron 5, rs144012689, was consistently present in all 9 *HLA-B^∗^15:02* subtypes and in only one other *HLA-B* allele, *HLA-B^∗^15:13*. *HLA-B^∗^15:13* is not associated with SJS/TEN or CBZ side effects in any ethnicity. The minor allele of rs144012689 (“T” on the minus strand) was found in *HLA-B^∗^15:02* (including its subtypes) and *HLA-B^∗^15:13*, while all other *HLA-B* references carried the major allele (“A” on the minus strand).

To test the hypothesis that rs144012689 could be a genetic marker for *HLA-B^∗^15:02*, we checked the allele frequencies of rs144012689 minor allele, *HLA-B^∗^15:02, and HLA-B^∗^15:13* in major ethnic groups in public databases [1000 Genomes and the Allele Frequency Net Database (Gonzalez-Galarza et al., 2015), respectively; Table 1]. Comparable allele frequencies for rs144012689 minor allele and *HLA-B^∗^15:02* were observed in global populations (Asians, Caucasians, Africans, and Hispanics) in spite of a nearly 500–1000-fold difference in the number of subjects for some of the ethnic groups (Table 1). In contrast, *HLA-B^∗^15:13* was generally found at only about 1/10 of the allele frequency of *HLA-B^∗^15:02* in major ethnic groups (Table 1) based on the comprehensive HLA allele frequency database – the Allele Frequency Net Database (Gonzalez-Galarza et al., 2015). We therefore considered that rs144012689 could be a useful genetic marker for *HLA-B^∗^15:02*. In addition, a recent study in Hong Kong Chinese patients with drug-induced cutaneous reactions also identified rs144012689 as a highly sensitive and specific marker for *HLA-B^∗^15:02* (Gui et al., 2017).

**Table 1:** Global and population frequencies of rs144012689, *HLA-B^∗^15:02*, and *HLA-B^∗^15:13.*

Allele	Global Populations	African	Asian	Caucasian	Hispanic
rs144012689 MAF	1.8% (*n* = 5,008)	0% AFR (*n* = 1,322)	4.6% SAS, EAS (*n* = 1,986)	0% EUR (*n* = 1,066)	0% AMR (*n* = 694)
*HLA-B^∗^15:02*	0.91% (*n* = 2,453,203)	0.21% (*n* = 41,314)	3.92% (*n* = 560,842)	0.001% (*n* = 1,325,156)	0.03% (*n* = 417,406)
*HLA-B^∗^15:13*	0.08% (*n* = 2,145,939)	0.04% (*n* = 8,540)	0.43% (*n* = 353,749)	0.0002% (*n* = 1,288,839)	0% (*n* = 263,703)


*Data sources: www.allelefrequencies.net (HLA frequencies) and 1000 Genomes Project (rs144012689); MAF, minor allele frequency; AFR, African; SAS, South Asian; EAS, East Asian; EUR, European; AMR, ad-mixed American.*

### Concordance Between rs144012689 Minor Allele and *HLA-B^∗^15:02* in Samples From Reference Databases

We hypothesized that rs144012689 minor allele would be present in any sample in which *HLA-B^∗^15:02* was observed, and evaluated the concordance between the rs144012689 and *HLA-B^∗^15:02* by comparing HLA typing and the rs144012689 genotypes from public databases. rs144012689 genotypes and *HLA-B* typing results of the 1000 Genomes Project (Gourraud et al., 2014) samples were retrieved from 1000 Genomes and NCBI dbMHC, respectively. *HLA* typing was performed by standard Sanger sequencing (Gourraud et al., 2014). A full list of the 955 samples with both rs144012689 and *HLA-B* results is given in Supplementary Table S1. This reference sample group contained subjects from the following: African Ancestry (Southwest United States), Yoruba (Ibadan, Nigeria), Colombian (Medellin, Colombia), Mexican Ancestry (Los Angeles-California, United States), Puerto Rican (Puerto Rico), Northern and Western European (Utah, United States), Finnish (Finland), British (England and Scotland, United Kingdom), Italian (Tuscany, Italy), Han (Beijing and south, China), and Japanese (Tokyo, Japan).

*HLA-B^∗^15:02* genotyping results were considered “positive” if one or two copies of *HLA-B^∗^15:02* were present, or “negative” if no copies of *HLA-B^∗^15:02* were present. There was no intermediate genotype (Leckband et al., 2013; Saito et al., 2016). We found 100% concordance between rs144012689 minor allele carriers and *HLA-B^∗^15:02* positives: of the 955 samples, 12 *HLA-B^∗^15:02* positives also carried the minor allele of rs144012689 and the remaining 943 which were negative for *HLA-B^∗^15:02* did not (Table 2). There were no false positives (FP) or false negatives (FN). In this data set, the rs144012689 minor allele showed 100% concordance to the presence of *HLA-B^∗^15:02* (100% sensitivity and 100% specificity). The correlation was statistically significant (*P* < 0.0001).

**Table 2:** Correlation between rs144012689 minor allele and *HLA-B^∗^15:02* in 955 samples from public databases.

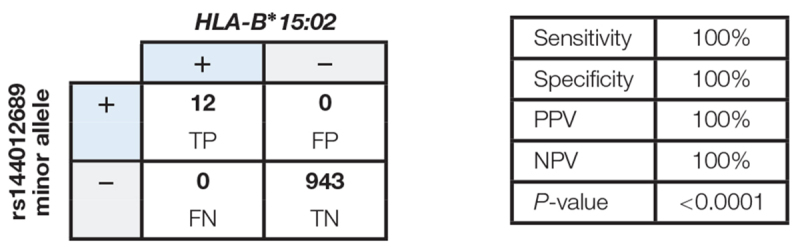

*955 samples from 1000 Genomes that were Sanger-sequenced for HLA-B were compared to determine whether rs144012689 could be used to indicate the presence of *HLA-B^∗^15:02*. TP (true positive), samples that were positive for both rs144012689 minor allele and *HLA-B^∗^15:02*; FP (false positive), samples that were positive for rs144012689 minor allele and were negative for *HLA-B^∗^15:02*; FN (false negative), samples that were negative for rs144012689 minor allele but positive for *HLA-B^∗^15:02*; TN (true negative), samples that were negative for rs144012689 minor allele and were also negative for *HLA-B^∗^15:02*; PPV, positive predictive value; NPV, negative predictive value; ^∗^*P*-value was calculated by Chi-square test. All 12 TP specimens were from the Han Chinese HapMap project (see Supplementary Table S1 for details). 943 TN were from the following: African Ancestry from Southwest, United States; British from England and Scotland, United Kingdom; Colombian from Medellin, Colombia; Finnish, Finland; Han from Beijing, China + Japanese from Tokyo, Japan; Han from South China; Italian from Tuscany, Italy; Luhya from Webuye, Kenya; Mexican Ancestry from Los Angeles; Northern and Western European from Utah, United States; Puerto Rican, Puerto Rico; Yoruba from Ibadan, Nigeria.*

### Association Between rs144012689 and *HLA-B^∗^15:02* in a Large Cohort of >28,000 United States Patients

Based on the accumulating evidence that rs144012689 may be a useful marker for *HLA-B^∗^15:02*, we undertook a study in a large patient cohort to compare *HLA-B* status with respect to rs144012689 genotype to determine whether the association held in a large sample set from the diverse United States population. Each sample was sequenced in exons 2/3 for *HLA-B* typing and intron 5 for rs144012689 genotyping. The cohort included 28,897 samples from United States patients whose physicians ordered pharmacogenetic testing from Millennium Health (San Diego, CA, United States), and was supplemented with samples selected based on known positive *HLA-B^∗^15:02* status. At the time of ordering the physician could optionally select patient ethnicity from the following: African-American, Asian, Caucasian, Hispanic, and Other. Results for rs144012689 were compared to *HLA-B* typing results.

The data showed a strong association between rs144012689 and *HLA-B^∗^15:02*. Among 28,897 samples sequenced, 51 were positive for both rs144012689 minor allele and *HLA-B^∗^15:02* (TP), and 28,838 were negative for both rs144012689 minor allele and *HLA-B^∗^15:02* (TN). There were no FN. By this evaluation we also observed eight false positive samples (positive for rs144012689 minor allele but negative for *HLA-B^∗^15:02*) which was due to the presence of *HLA-B^∗^15:13* in all 8 cases. Thus, in the cohort of nearly 30,000 subjects, rs144012689 minor allele showed 100% sensitivity and 99.97% specificity to detect *HLA-B^∗^15:02* (Table 3).

**Table 3:** Correlation of rs144012689 minor allele with *HLA-B^∗^15:02* allele in a large cohort of clinical samples.

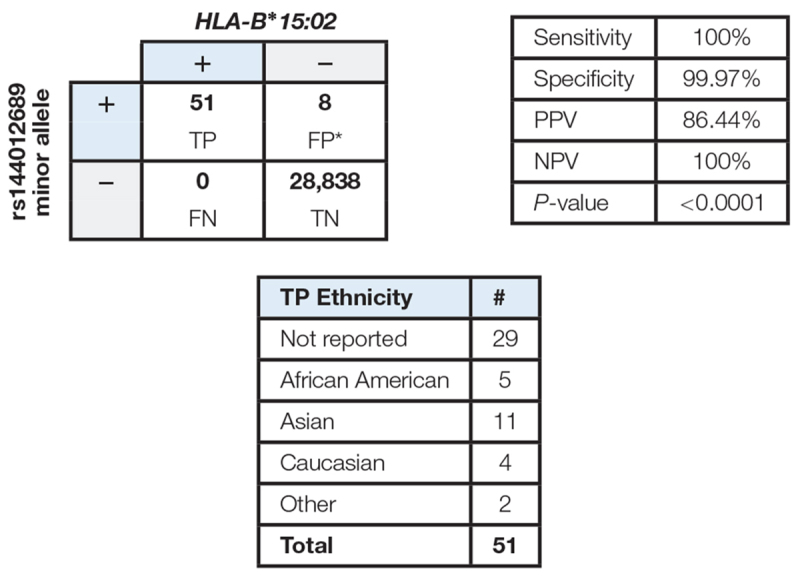

*28,897 samples were sequenced for HLA-B haplotype and intron 5 for rs144012689. TP (true positive), samples that were positive for both rs144012689 minor allele and *HLA-B^∗^15:02*; FP (false positive), samples that were positive for rs144012689 minor allele and negative for *HLA-B^∗^15:02*; FN (false negative), samples that were negative for rs144012689 minor allele but positive for *HLA-B^∗^15:02*; TN (true negative), samples that were negative for rs144012689 minor allele and were also negative for *HLA-B^∗^15:02*; PPV, positive predictive value; NPV, negative predictive value; *P*-value was calculated by Chi-square test. The ethnic breakdown of the TP samples is shown. ^∗^All FP samples were *HLA-B^∗^15:13*.*

### Ability to Detect rs144012689 by PCR

Given the strong association between rs144012689 and *HLA-B^∗^15:02*, and in order to create an effective screening test for *HLA-B^∗^15:02*, we developed a PCR-based genotyping assay for rs144012689. To demonstrate the accuracy of the PCR-based assay we selected an enriched cohort of known samples containing a variety of genotypes for rs144012689 (total *n* = 1,122). This set was comprised of 32 *HLA-B^∗^15:02* positive reference samples and 1,090 additional samples including 38 carrying *HLA-B^∗^15:02* and 8 carrying *HLA-B^∗^15:13*. These samples were sequenced for *HLA-B* and rs144012689 and in parallel were tested by PCR-based genotyping. The *HLA-B* and rs144012689 results of the 32 control *HLA-B^∗^15:02* positive cell line controls are listed in Supplementary Table S2.

rs144012689 PCR results showed 100% agreement to intron 5 sequence data for all 1,122 samples for the three possible genotypes: T/T (*n* = 3), A/T (*n* = 75), and A/A (*n* = 1044). Interpreting rs144012689 as a genetic marker for *HLA-B^∗^15:02* in this selected cohort gives 70 TP, 1,044 TN, 8 FP, and 0 FN, which obtains 100% sensitivity and 99.2% specificity to detect *HLA-B^∗^15:02*. In this study we also identified a novel allele not in the databases that carries rs144012689, *HLA-B^∗^15:XXX*. This novel allele was confirmed to carry the rs144012689 T allele by NGS. The sequence data show it has a non-synonymous mutation in exon-2 at AA-65 CAG(Q) – >CAC (H). Thus, the PCR assay provides 100% accuracy for genotyping of rs144012689 as compared to sequencing.

### Allele Frequency and Ethnicity Information for rs144012689 Minor Allele in a Large Patient Cohort

We next evaluated the frequency of *HLA-B^∗^15:02* and rs144012689 in a larger cohort (130,460 subjects) of which 35,710 were sequenced for *HLA-B* (inclusive of the 28,897 samples mentioned previously) and the remaining 94,750 were genotyped for rs144012689. The positivity rate in the group sequenced for *HLA-B^∗^15:02* was 32/35,710 (0.090%); in the genotyped group the positivity rate for rs144012689 was 121/94,750 (0.128%). In the sequenced group, only *HLA-B^∗^15:02* positives are represented; in the genotyped group *HLA-B^∗^15:02* samples and any carriers of *HLA-B^∗^15:13* or other unknown novel alleles bearing rs144012689 minor allele would also be present. The proportion of *HLA-B^∗^15:13* is expected to be about 1/10 the frequency of *HLA*-*B^∗^15:02* in all populations (see Table 1).

**FIGURE 1:**
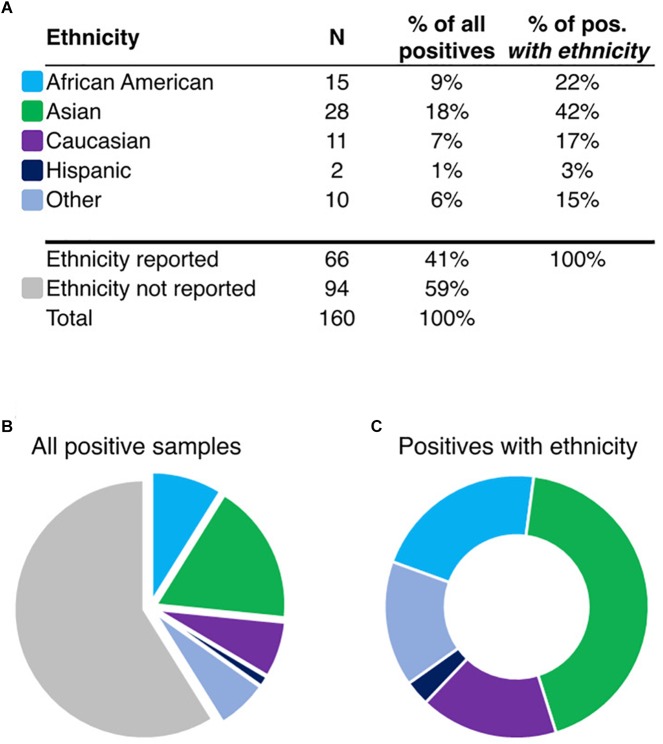
Ethnicity information for rs144012689-positive patients (**A**, *N* = 160). Pie charts show the proportion of samples reported in ethnic groups in the total set **(B)**, or in the ethnicity-reported subset (**C**, *N* = 66). “Other” includes the following: Asian/Caucasian, Asian/Hispanic, Hawaiian, Indian, and Other. Colors shown in **(B,C)** are identified in **(A)** by ethnicity.

To determine the ethnic frequency of samples positive for *HLA-B^∗^15:02* in the tested population (*N* = 130,460) using rs144012689 as a proxy for *HLA-B^∗^15:02*, we compared physician-reported ethnicity in samples carrying the minor allele as determined by direct *HLA-B* intron 5 sequencing or by rs144012689 genotyping. Among 160 subjects positive for rs144012689, ethnicity was reported for 66 of them (Figure 1A,B). Interestingly, 28 of those 66 patients were reported as Asian (42%) while the rest were African American (*N* = 15, 23%), Caucasian (*N* = 11, 17%), Hispanic (*N* = 2, 3%), and Other (*N* = 10, 15%) (Figure 1C). 51 samples in this cohort were sequenced and verified as *HLA-B^∗^15:02* positives, and of these, 23 had reported ethnicity. Similar ethnic distribution was observed; 11 of those 23 were reported as Asian (48%), and the rest were African American (*N* = 5, 22%), Caucasian (*N* = 4, 17%), and Other (*N* = 3, 13%).

## Discussion

Pharmacogenetic testing provides physicians and patients with a useful tool to aid in optimizing dosing, identifying potential safety risks and indicating likelihood of efficacy associated with patient genotypes for related medications. It is known that in certain Asian populations, *HLA-B* gene status is a critical factor for safe prescribing of CBZ and its analogs due to the prevalence of the *HLA-B^∗^15:02* allele and its association with risk of severe adverse events including SJS and TEN. The importance of pre-prescription genetic testing is recognized. FDA recommends genetic testing for *HLA-B^∗^15:02* in patients with Asian ancestry prior to prescribing CBZ (FDA, 2015). In response, Medicare coverage for *HLA-B^∗^15:02* testing prior to CBZ therapy has also been limited to those with Asian and Oceanian ancestry. *HLA-B^∗^15:02* pharmacogenetic testing is routine in countries like Taiwan (Chen et al., 2011) where the allele frequency of this allele is appreciable (e.g., the carrier rate is ∼7% in China South Han, ∼6–18% in Taiwan, ∼8% in Thailand, and ∼6–12% in Singapore) (Farkas, 2009; Gonzalez-Galarza et al., 2015).

While it is possible to do full *HLA-B* genotyping and determine the specific alleles to various degrees of resolution by sequencing, for the purposes of screening *HLA-B^∗^15:02* it is necessary only to identify potential carriers of the risk allele out of the alleles possible. In this case, sensitive detection of the allele of interest is of greater importance than the identification of other alleles that may be present. In fact, it has been suggested in a retrospective study performed in Thailand that an economic benefit would be realized if a test specific for *HLA-B^∗^15:02* rather than full *HLA-B* typing were performed in that population (Locharernkul et al., 2010). Furthermore, in a prospective study in Taiwan (4,877 patients), pre-prescription screening for *HLA-B^∗^15:02* and subsequent medication recommendations for *HLA-B^∗^15:02* positives led to a reduction to zero in the number of SJS/TEN cases as compared to historical incidence expected for the number of patients in the study (Chen et al., 2011).

The *HLA* genes are among the most polymorphic in the human genome (Leckband et al., 2013). Pairing this genomic complexity with variable ethnic frequencies makes a challenging target for which to create a standardized test. An optimal screening test would be one that provides high accuracy, sensitivity and specificity, paired with low cost and fast turnaround. We set out to identify such a method to screen for *HLA-B^∗^15:02* and show that rs144012689 in *HLA-B* intron 5 is a strongly associated genetic marker for *HLA-B^∗^15:02*. In our cohort of nearly 30,000 subjects, we demonstrated that rs144012689 was a reliable genetic marker for *HLA-B^∗^15:02*, with 100% sensitivity and 99.97% specificity and PPV of 86.44% (due to the expected co-detection of *HLA-B^∗^15:13)*. Importantly, all subjects bearing *HLA-B^∗^15:02* were correctly detected by rs144012689. The absence of false negative results is of critical importance, as any patient falsely identified as *HLA-B^∗^15:02-*negative could be prescribed CBZ and thus could be at risk of SJS/TEN or other adverse effects.

Since rs144012689 is located within the *HLA-B* gene, its use as a genetic marker for *HLA-B^∗^15:02* is advantageous over conventional tagging or proxy SNPs which are found in linkage disequilibrium (LD) with a certain haplotype but are not part of the gene itself. Particularly, a two-SNP haplotype consisting of the minor alleles of rs2844682 and rs3909184 has been reported to tag the *HLA-B^∗^15:02* allele in 45 unrelated individuals from the HapMap (International HapMap Consortium, 2003) population of Han Chinese in Beijing, China (CHB) (de Bakker et al., 2006). However, recent studies have shown these two SNPs do not accurately tag *HLA-B^∗^15:02.* In two independent studies these SNPs showed sensitivity of 6 and 31.8% (He et al., 2015; Zhu et al., 2015). Another recent report shows a linkage of rs10484555 to *HLA-B^∗^15:02* with 100% sensitivity and nearly 100% specificity in 1,380 Han Chinese (Liu et al., 2015). It is important to note that this discovery is population-specific and the LD between *HLA-B^∗^15:02* and this tag SNP in the Han Chinese population may be different in other populations. An important finding of this study is that rs144012689 was able to identify *HLA-B^∗^15:02* in individuals of any background, regardless of ethnicity.

One of the most striking findings in our study was the presence of *HLA-B^∗^15:02* in patients who were reported to be of non-Asian ethnicities. More than half of positives in our cohort were identified with ethnicity other than Asian. Emerging evidence has also shown that patients in other regions may also carry the allele and suffer from adverse effects on CBZ therapy, due to migration, admixture, or previously un-appreciated population-based carriership. For example, in a European study of 12 German and French patients with SJS, four of the patients actually had Asian ancestry and were found to carry *HLA-B^∗^15:02* (Lonjou et al., 2006). In a Spanish Romani patient not of Asian ancestry, *HLA-B^∗^15:02* was found after the patient developed SJS on CBZ therapy, representing the first published case of a person assumed not to be Asian with SJS associated with the allele (Bellon et al., 2016). The authors subsequently identified that the Romani population has a 1% allele frequency with a potentially more ancestral connection to South Asia. A central conclusion of Lonjou et al. (2006) is that ethnicity matters for assessing risk of adverse drug reactions from CBZ in *HLA-B^∗^15:02* carriers. Our study is in agreement with this conclusion, and additionally highlights that in United States patients, factors such as migration and admixture may complicate the ability to assess genetic ethnicity based risk without more sophisticated tools such as empirical determination of genetic ethnicity or direct testing for risk factors such as the *HLA-B^∗^15:02* allele.

For the prescribing physician, determining genetic-ethnicity-based risk may be challenging. Patients may be unaware of the relevance of their Asian ancestry or fail to inform their doctors of their heritage (Barbarino et al., 2015). Studies of ancestral admixture analysis paired with self-declared ethnicity have shown that in general self-reporting correlates well with overall genetic ethnicity, but that in itself is not necessarily a good indicator of individual ancestry (Guthery et al., 2007). Each ethnic group is comprised of significant admixture when ancestry informative markers in autosomal DNA are considered (Lao et al., 2010), which leaves an important gap in knowledge about the extent of admixture and the specifics of which portions of the genome are derived from which ancestral contributors. The Clinical Pharmacology Implementation Consortium (CPIC) has also recognized this and has indicated it may be important to consider *HLA-B^∗^15:02* status when prescribing CBZ, regardless of self-reported ethnicity (Leckband et al., 2013).

These findings, combined with our data showing that patients of physician-reported non-Asian ethnicity made up more than half of the positives in our analysis of 130,460 subjects, indicates that basing the decision to do genetic testing only on those of declared Asian ancestry would be insufficient to identify more than half of positives in our United States cohort. Since Asian ethnicity is a key indicator for genetic testing before CBZ therapy according to FDA labeling guidelines and insurance coverage, our results suggest that the current screening recommendations may fail to detect *HLA-B^∗^15:02* in a number of United States patients, leading to the potential for increased risk of adverse events by CBZ. A study of the clinical utility of *HLA-B^∗^15:02* screening in non-Asian patients may be warranted to extend our understanding of the impact of these findings.

Limitations of this analysis include the fact that patient ethnicity is reported in an uncontrolled fashion; physicians may obtain ethnicity by their own observation, by requesting the patient to self-report, or other unknown means. Ethnicity is not reported for all patients. We and others have noted that physicians may not select ethnicity on a laboratory test order form when it is optional (Beoris et al., 2016; Del Tredici et al., 2018). Patients may not accurately know or may fail to identify their ethnicity correctly. Patients reporting more than one ethnicity may be classified as “Other.” *HLA-B* data was obtained through routine panel testing and was not necessarily part of planned CBZ therapy. There are two known FP using this SNP as a marker for *HLA-B^∗^15:02*, both of which are at a much lower allele frequency than *HLA-B^∗^15:02* itself [*HLA-B^∗^15:13* (see Supplementary Table S2) and *HLA-B^∗^15:XXX* (detected by NGS; see Results)].

In summary, here we describe an accurate method to screen for *HLA-B^∗^15:02* using a SNP located within the *HLA-B* gene. The SNP shows 100% sensitivity and 99.97% specificity in our large patient cohort. Our results suggest that screening for *HLA-B^∗^15:02* based on observed or self-reported Asian ancestry may be insufficient to identify potential carriers of the allele in the ethnically diverse United States population.

## Author Contributions

HF participated in the conception and design, data collection and analysis, and drafting the manuscript. XX participated in the design and bioinformatics analysis. KK participated in the conception and design, data analysis and interpretation, and critical revision. MD participated in the conception and design, analysis, and interpretation. GZ participated in data analysis and critical revision. BR and FE participated in the data collection and analysis. ADT participated in interpretation, drafting, and critical revision of the manuscript. TM participated in the conception, design, analysis, interpretation, drafting, and revising the manuscript.

## Conflict of Interest Statement

All authors were full-time employees of Millennium Health, San Diego, CA, United States during the course of this study. In addition, United States Patent 9932638: Single nucleotide polymorphism in *HLA-B^∗^15:02* and use thereof with authors HF, KK, MD, XX, and TM has been issued.

## Footnotes

1 http://hla.alleles.org/nomenclature/stats.html

2 http://hla.alleles.org/data/txt/b_gen.txt

3 http://browser.1000genomes.org

4 http://www.ncbi.nlm.nih.gov/projects/gv/mhc/xslcgi.fcgi?cmd=cellsearch
