# Supplementary material for: A Screening Test for HLA-B∗15:02 in a Large United States Patient Cohort Identifies Broader Risk of Carbamazepine-Induced Adverse Events
Source: Front Pharmacol. 2019 Mar 26;10:149. doi: 10.3389/fphar.2019.00149 (PMC6443844; doi:10.3389/fphar.2019.00149)
Supplement: Supplementary file 1 [file Data_Sheet_1.docx]

Supplementary Material

**A Novel Screening Test for *HLA-B*15:02* in a Large US Patient Cohort Identifies Broader Risk of Carbamazepine-Induced Adverse Events**

**Hua Fang, PhD, Xiequn Xu, PhD, Kulvinder Kaur, DPhil, Matthew Dedek, MS, Guang-dan Zhu, PhD, Bae J. Riley, CGMBS, Frank G. Espin, CGMBS, Andria L. Del Tredici, PhD, and Tanya A. Moreno, PhD***

*** Correspondence:** Dr. Tanya A. Moreno, PhD, [tanya.moreno@millenniumhealth.com](mailto:tanya.moreno@millenniumhealth.com)

**List of Supplementary Content**:

Table S1: Supplementary Table 1.xlsx

Table S2: Supplementary Table 2.xlsx

Figure S1: Supplementary Figure 1.ppt

**Supplementary Figure Legends:**

**Table S1**: 955 samples from 1000 Genomes project with full *HLA-B* typing results and rs144012689 genotypes. Sample ID and gender are listed in column 1. Population from which each sample was derived is indicated. *HLA-B* alleles shown (allele 1 and allele 2) were typed as described in Gourraud et al, 2017 (manuscript ref 18).

**Table S2**: *HLA-B* alleles and rs144012689 genotypes for 32 positive samples selected for the PCR vs sequencing study. *HLA-B* alleles shown were typed by Histogenetics; PCR genotypes were derived in this study.

**Figure S1**: Representative scatter plot of rs144012689 (Fang et al., 2015). X axis = VIC, Y axis = FAM. NTC = no template control. Clusters indicated by color and *HLA-B*15:02* status. In this example run, 32 *HLA-B*15:02*-positive cell line control samples plus 56 *HLA-B*15:02-*negative patient samples were tested. Three positive homozygote samples (NA23093 with genotype of *HLA-B*15:02/15:02*, NA18122 with genotype of HLA-*B*15:02/15:02*, and IHW09199 with genotype of *HLA-B*15:02/15:13*) clustered as homozygous for minor allele ‘T’. All remaining *HLA-B*15:02*-positive samples were in the heterozygous cluster, and all the *HLA-B*15:02* negative samples clustered in the major allele homozygote.
