# Supplementary figures and images for: A Screening Test for HLA-B∗15:02 in a Large United States Patient Cohort Identifies Broader Risk of Carbamazepine-Induced Adverse Events
Source: Front Pharmacol. 2019 Mar 26;10:149. doi: 10.3389/fphar.2019.00149 (PMC6443844; doi:10.3389/fphar.2019.00149)

**Supplementary Figure S1.** Representative genotyping results for rs144012689 RT-PCR genotyping assay

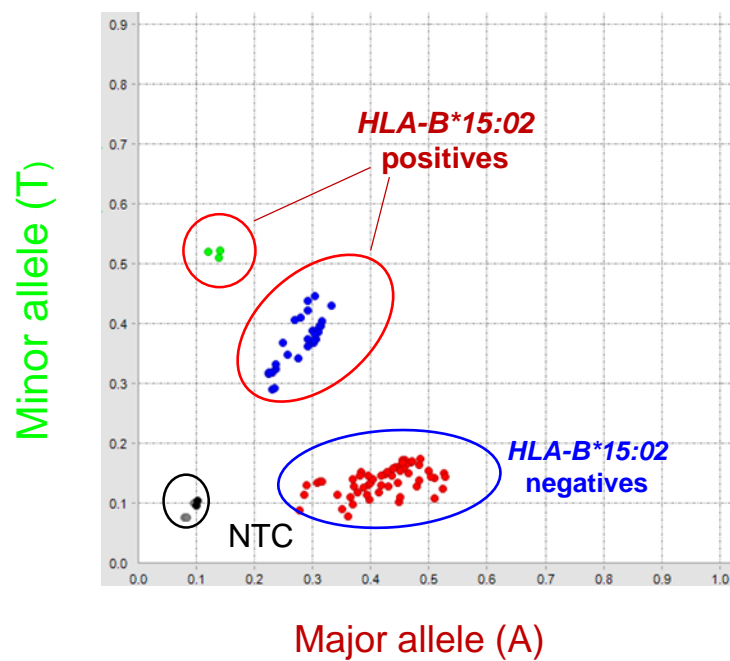

Supplement: Supplementary file 2 [file Image_1.pdf]
