# Supplementary material for: A Screening Test for HLA-B∗15:02 in a Large United States Patient Cohort Identifies Broader Risk of Carbamazepine-Induced Adverse Events
Source: Front Pharmacol. 2019 Mar 26;10:149. doi: 10.3389/fphar.2019.00149 (PMC6443844; doi:10.3389/fphar.2019.00149)
Supplement: Supplementary file 3 [file Table_1.pdf]

**Individual** (1000 Genomes Project Phase 3 Naming)	**rs144012689 Genotype** (minus strand;1000 Genomes)	**Population**	***HLA-B* Allele 1** (from NCBI dbMHC portal)	***HLA-B* Allele 2** (from NCBI dbMHC portal)
NA19625 (F)	A|A	1000 Genomes - African Ancestry from Southwest, USA	07:02:01/07:02:06/07:02:09/07:44/07:49N/07:58/07:59/07:61	44:03:02
NA19700 (M)	A|A		15:10	42:01:00
NA19701 (F)	A|A		15:37	40:02:01/40:56/40:97
NA19703 (M)	A|A		44:02:01:01/44:02:01:02S/44:19N/44:27/44:66	58:01:01/58:11
NA19704 (F)	A|A		15:10	51:01:01/51:01:05/51:01:07/51:11N/51:30/51:32/51:48/51:51
NA19707 (F)	A|A		07:02:01/07:02:06/07:02:09/07:44/07:49N/07:58/07:59/07:61	40:01:01/40:01:02/40:55
NA19711 (M)	A|A		35:01:01/35:01:03/35:40N/35:42/35:57/35:94	53:01:01
NA19712 (F)	A|A		15:03:01/15:103	45:01/45:07
NA19713 (F)	A|A		13:02:01/13:02:05	45:01/45:07
NA19818 (M)	A|A		39:10:00	42:01:00
NA19819 (F)	A|A		40:01:01/40:01:02/40:55	58:02:00
NA19834 (M)	A|A		35:01:01/35:01:03/35:40N/35:42/35:57/35:94	39:10:00
NA19835 (F)	A|A		50:01:00	53:01:01
NA19900 (M)	A|A		42:01:00	44:03:01/44:03:03/44:03:04
NA19901 (F)	A|A		39:06:02	58:02:00
NA19904 (M)	A|A		35:03:01/35:70	44:02:01:01/44:02:01:02S/44:19N/44:27/44:66
NA19908 (M)	A|A		50:01:00	58:01:01/58:11
NA19909 (F)	A|A		42:01:00	42:01:00
NA19914 (F)	A|A		35:01:01/35:01:03/35:40N/35:42/35:57/35:94	44:03:01/44:03:03/44:03:04
NA19916 (M)	A|A		15:03:01/15:103	53:01:01
NA19917 (F)	A|A		15:03:01/15:103	41:02:01
NA19920 (M)	A|A		08:01:01/08:19N	57:03:01
NA19921 (F)	A|A		15:01:01:01/15:01:01:02N/15:01:06/15:01:07/15:102/15:104/15:140/15:146	48:01:01/48:09
NA19982 (M)	A|A		07:02:01/07:02:06/07:02:09/07:44/07:49N/07:58/07:59/07:61	27:05:02/27:05:04/27:13
NA20126 (M)	A|A		27:05:02/27:05:04/27:13	57:03:01
NA20127 (F)	A|A		13:02:01/13:02:05	35:01:01/35:01:03/35:40N/35:42/35:57/35:94
NA20276 (F)	A|A		44:03:01/44:03:03/44:03:04	49:01:01
NA20278 (M)	A|A		44:03:01/44:03:03/44:03:04	49:01:01
NA20281 (M)	A|A		15:17:01:01/15:17:01:02	53:01:01
NA20282 (F)	A|A		53:01:01	81:01/81:02/81:03
NA20287 (F)	A|A		15:03:01/15:103	55:01:01/55:01:03
NA20289 (F)	A|A		42:01:00	53:01:01
NA20291 (M)	A|A		14:01	58:01:01/58:11
NA20294 (F)	A|A		13:02:01/13:02:05	45:01/45:07
NA20296 (F)	A|A		44:03:01/44:03:03/44:03:04	58:01:01/58:11
NA20299 (F)	A|A		35:01:01/35:01:03/35:40N/35:42/35:57/35:94	44:05:00
NA20314 (F)	A|A		07:02:01/07:02:06/07:02:09/07:44/07:49N/07:58/07:59/07:61	51:01:01/51:01:05/51:01:07/51:11N/51:30/51:32/51:48/51:51
NA20317 (F)	A|A		58:01:01/58:11	58:01:01/58:11
NA20332 (F)	A|A		07:02:01/07:02:06/07:02:09/07:44/07:49N/07:58/07:59/07:61	53:01:01
NA20334 (F)	A|A		07:02:01/07:02:06/07:02:09/07:44/07:49N/07:58/07:59/07:61	53:01:01
NA20340 (M)	A|A		15:16	44:03:01/44:03:03/44:03:04
NA20342 (M)	A|A		53:01:01	53:01:01
NA20346 (M)	A|A		15:10	53:01:01
NA20348 (M)	A|A		18:01:01/18:01:03/18:17N	42:01:00
NA20356 (M)	A|A		07:02:01/07:02:06/07:02:09/07:44/07:49N/07:58/07:59/07:61	78:01:00
NA20357 (F)	A|A		07:02:01/07:02:06/07:02:09/07:44/07:49N/07:58/07:59/07:61	81:01/81:02/81:03
NA20359 (F)	A|A		53:01:01	53:01:01
NA18501 (M)	A|A		14:01	78:01:00
NA18502 (F)	A|A		14:03	58:01:00
NA18504 (M)	A|A		15:03	39:10:00
NA18505 (F)	A|A		15:03	53:01:00
NA18507 (M)	A|A		15:03	42:01:00
NA18508 (F)	A|A		42:01:00	53:01:00
NA18516 (M)	A|A		15:10	53:01:00
NA18517 (F)	A|A		49:01:00	51:01:00
NA18522 (M)	A|A		15:03	49:01:00

**Individual** (1000 Genomes Project Phase 3 Naming)	**rs144012689 Genotype** (minus strand;1000 Genomes)	**Population**	***HLA-B* Allele 1** (from NCBI dbMHC portal)	***HLA-B* Allele 2** (from NCBI dbMHC portal)
NA18523 (F)	A|A	1000 Genomes - Yoruba from Ibadan, Nigeria	7:02	35:01:00
NA18853 (M)	A|A		44:03:01/44:03:03/44:03:04	52:01:00
NA18856 (M)	A|A		35:01:00	49:01:00
NA18858 (F)	A|A		18:01	49:01:00
NA18861 (F)	A|A		13:02:01/13:02:05	57:03:00
NA18870 (F)	A|A		15:03	57:03:00
NA18871 (M)	A|A		42:01:00	52:01:00
NA18912 (F)	A|A		53:01:00	53:01:00
NA19092 (M)	A|A		42:01:00	81:01:00
NA19093 (F)	A|A		35:01:00	53:01:00
NA19098 (M)	A|A		51:01:00	53:01:00
NA19099 (F)	A|A		7:02	7:02
NA19102 (F)	A|A		18:01	42:01:00
NA19119 (M)	A|A		35:01:01/35:01:03/35:40N/35:42/35:57/35:94	49:01:01
NA19129 (F)	A|A		7:02	58:01:00
NA19130 (M)	A|A		13:02:01/13:02:05	35:01:00
NA19131 (F)	A|A		42:01:00	53:01:00
NA19137 (F)	A|A		7:02	45:01:00
NA19138 (M)	A|A		7:02	58:01:00
NA19141 (M)	A|A		35:01:00	52:01:00
NA19143 (F)	A|A		52:01:00	53:01:00
NA19144 (M)	A|A		53:01:00	58:01:00
NA19152 (F)	A|A		15:10	56:01:00
NA19153 (M)	A|A		18:01	53:01:00
NA19159 (F)	A|A		52:01:00	57:02:00
NA19160 (M)	A|A		15:10	51:01:00
NA19171 (M)	A|A		52:01:00	53:01:00
NA19172 (F)	A|A		35:01:00	44:03:01/44:03:03/44:03:04
NA19200 (M)	A|A		7:02	52:01:00
NA19201 (F)	A|A		15:16	18:01
NA19204 (F)	A|A		18:01	58:01:00
NA19206 (F)	A|A		42:01:00	53:01:00
NA19207 (M)	A|A		8:01	15:10
NA19209 (F)	A|A		15:10	52:01:00
NA19210 (M)	A|A		15:10	58:01:01/58:11
NA19222 (F)	A|A		42:02:00	45:01:00
NA19223 (M)	A|A		41:04:00	42:01:00
NA19238 (F)	A|A		53:01:00	57:03:00
NA19239 (M)	A|A		35:01:00	52:01:00
NA19027 (M)	A|A		53:01:01	57:03:01
NA19028 (M)	A|A		13:02:01/13:02:05	15:03:01/15:103
NA19031 (M)	A|A		42:01:00	53:01:01
NA19035 (M)	A|A		42:01:00	44:03:01/44:03:03/44:03:04
NA19036 (F)	A|A		53:01:01	58:02:00
NA19038 (F)	A|A		15:03:01/15:103	35:01:01/35:01:03/35:40N/35:42/35:57/35:94
NA19041 (M)	A|A		45:01/45:07	58:02:00
NA19307 (M)	A|A		52:01:01/52:07	81:01/81:02/81:03
NA19308 (M)	A|A		18:03	58:01:01/58:11
NA19309 (M)	A|A		27:03:00	58:02:00
NA19310 (F)	A|A		35:01:01/35:01:03/35:40N/35:42/35:57/35:94	53:01:01
NA19314 (F)	A|A		18:01:01/18:01:03/18:17N	51:01:01/51:01:05/51:01:07/51:11N/51:30/51:32/51:48/51:51
NA19315 (F)	A|A		45:01/45:07	58:01:01/58:11
NA19316 (F)	A|A		15:03:01/15:103	58:01:01/58:11
NA19317 (M)	A|A		15:03:01/15:103	42:01:00
NA19318 (M)	A|A		07:02:01/07:02:06/07:02:09/07:44/07:49N/07:58/07:59/07:61	82:02:00
NA19319 (M)	A|A		15:03:01/15:103	53:01:01

**Individual** (1000 Genomes Project Phase 3 Naming)	**rs144012689 Genotype** (minus strand;1000 Genomes)	**Population**	***HLA-B* Allele 1** (from NCBI dbMHC portal)	***HLA-B* Allele 2** (from NCBI dbMHC portal)
NA19321 (F)	A|A	1000 Genomes - Luhya from Webuye, Kenya	45:01/45:07	53:01:01
NA19324 (F)	A|A		08:01:01/08:19N	58:01:01/58:11
NA19327 (F)	A|A		45:01/45:07	45:01/45:07
NA19328 (F)	A|A		18:01:01/18:01:03/18:17N	53:01:01
NA19332 (F)	A|A		15:10	58:02:00
NA19334 (M)	A|A		15:10	45:01/45:07
NA19346 (M)	A|A		15:03:01/15:103	18:01:01/18:01:03/18:17N
NA19347 (M)	A|A		07:02:01/07:02:06/07:02:09/07:44/07:49N/07:58/07:59/07:61	58:01:01/58:11
NA19350 (M)	A|A		18:01:01/18:01:03/18:17N	58:02:00
NA19360 (M)	A|A		53:01:01	58:02:00
NA19372 (M)	A|A		51:01:01/51:01:05/51:01:07/51:11N/51:30/51:32/51:48/51:51	53:01:01
NA19374 (M)	A|A		35:01:01/35:01:03/35:40N/35:42/35:57/35:94	41:01:00
NA19375 (M)	A|A		15:10	15:03:01/15:103
NA19376 (M)	A|A		15:17:01:01/15:17:01:02	58:02:00
NA19377 (F)	A|A		42:01:00	82:02:00
NA19379 (F)	A|A		51:01:01/51:01:05/51:01:07/51:11N/51:30/51:32/51:48/51:51	53:01:01
NA19380 (M)	A|A		45:01/45:07	51:01:01/51:01:05/51:01:07/51:11N/51:30/51:32/51:48/51:51
NA19383 (M)	A|A		15:10	53:01:01
NA19384 (M)	A|A		35:01:01/35:01:03/35:40N/35:42/35:57/35:94	45:01/45:07
NA19385 (M)	A|A		08:01:01/08:19N	81:01/81:02/81:03
NA19390 (F)	A|A		15:03:01/15:103	48:05:00
NA19391 (F)	A|A		53:01:01	57:03:01
NA19393 (M)	A|A		15:17:01:01/15:17:01:02	15:03:01/15:103
NA19394 (M)	A|A		13:02:01/13:02:05	14:02:01
NA19397 (M)	A|A		08:01:01/08:19N	58:02:00
NA19399 (F)	A|A		18:01:01/18:01:03/18:17N	58:02:00
NA19403 (F)	A|A		35:01:01/35:01:03/35:40N/35:42/35:57/35:94	44:03:01/44:03:03/44:03:04
NA19404 (F)	A|A		53:01:01	58:02:00
NA19428 (M)	A|A		18:01:01/18:01:03/18:17N	53:01:01
NA19429 (M)	A|A		15:03:01/15:103	41:01:00
NA19430 (M)	A|A		39:10:00	58:01:01/58:11
NA19431 (F)	A|A		15:10	18:01:01/18:01:03/18:17N
NA19434 (F)	A|A		81:01/81:02/81:03	81:01/81:02/81:03
NA19435 (F)	A|A		15:10	42:01:00
NA19436 (F)	A|A		15:10	58:02:00
NA19437 (F)	A|A		18:01:01/18:01:03/18:17N	40:12:00
NA19438 (F)	A|A		49:01:01	58:01:01/58:11
NA19439 (F)	A|A		42:01:00	58:02:00
NA19440 (F)	A|A		42:01:00	45:01/45:07
NA19443 (M)	A|A		08:01:01/08:19N	53:01:01
NA19445 (F)	A|A		15:03:01/15:103	42:01:00
NA19446 (F)	A|A		45:01/45:07	51:01:01/51:01:05/51:01:07/51:11N/51:30/51:32/51:48/51:51
NA19448 (M)	A|A		58:02:00	81:01/81:02/81:03
NA19449 (F)	A|A		57:02:00	81:01/81:02/81:03
NA19451 (M)	A|A		51:01:01/51:01:05/51:01:07/51:11N/51:30/51:32/51:48/51:51	58:01:01/58:11
NA19452 (M)	A|A		15:03:01/15:103	53:01:01
NA19455 (M)	A|A		45:01/45:07	81:01/81:02/81:03
NA19456 (F)	A|A		53:01:01	58:01:01/58:11
NA19457 (F)	A|A		08:01:01/08:19N	81:01/81:02/81:03
NA19462 (F)	A|A		42:01:00	45:01/45:07
NA19463 (F)	A|A		53:01:01	58:01:01/58:11
NA19466 (M)	A|A		45:01/45:07	57:03:01
NA19467 (F)	A|A		53:01:01	53:01:01
NA19468 (F)	A|A		81:01/81:02/81:03	81:01/81:02/81:03
NA19471 (F)	A|A		53:01:01	73:01:00
NA19472 (F)	A|A		45:01/45:07	45:01/45:07

**Individual** (1000 Genomes Project Phase 3 Naming)	**rs144012689 Genotype** (minus strand;1000 Genomes)	**Population**	***HLA-B* Allele 1** (from NCBI dbMHC portal)	***HLA-B* Allele 2** (from NCBI dbMHC portal)
NA19473 (F)	A|A		58:02:00	73:01:00
NA19474 (F)	A|A		45:01/45:07	45:01/45:07
HG01112 (M)	A|A	1000 Genomes - Colombian from Medellin, Colombia	38:01:01	44:02:01:01/44:02:01:02S/44:19N/44:27/44:66
HG01113 (F)	A|A		35:43/35:67/35:79	39:11:00
HG01124 (M)	A|A		40:04:00	40:04:00
HG01125 (F)	A|A		15:01:01:01/15:01:01:02N/15:01:06/15:01:07/15:102/15:104/15:140/15:146	35:12:00
HG01133 (M)	A|A		08:01:01/08:19N	35:01:01/35:01:03/35:40N/35:42/35:57/35:94
HG01134 (F)	A|A		39:08:00	57:01:01
HG01136 (M)	A|A		35:01:01/35:01:03/35:40N/35:42/35:57/35:94	53:01:01
HG01137 (F)	A|A		07:02:01/07:02:06/07:02:09/07:44/07:49N/07:58/07:59/07:61	15:01:01:01/15:01:01:02N/15:01:06/15:01:07/15:102/15:104/15:140/15:146
HG01139 (M)	A|A		41:02:01	50:01:00
HG01140 (F)	A|A		39:11:00	44:03:01/44:03:03/44:03:04
HG01148 (M)	A|A		07:02:01/07:02:06/07:02:09/07:44/07:49N/07:58/07:59/07:61	35:08:01
HG01149 (F)	A|A		35:43/35:67/35:79	44:02:01:01/44:02:01:02S/44:19N/44:27/44:66
HG01250 (M)	A|A		35:12:00	35:43/35:67/35:79
HG01251 (F)	A|A		35:43/35:67/35:79	49:01:01
HG01253 (M)	A|A		14:02:01	27:05:02/27:05:04/27:13
HG01254 (F)	A|A		07:02:01/07:02:06/07:02:09/07:44/07:49N/07:58/07:59/07:61	44:02:01:01/44:02:01:02S/44:19N/44:27/44:66
HG01256 (M)	A|A		07:02:01/07:02:06/07:02:09/07:44/07:49N/07:58/07:59/07:61	15:01:01:01/15:01:01:02N/15:01:06/15:01:07/15:102/15:104/15:140/15:146
HG01257 (F)	A|A		07:02:01/07:02:06/07:02:09/07:44/07:49N/07:58/07:59/07:61	18:01:01/18:01:03/18:17N
HG01259 (M)	A|A		40:01:01/40:01:02/40:55	51:01:01/51:01:05/51:01:07/51:11N/51:30/51:32/51:48/51:51
HG01260 (F)	A|A		07:02:01/07:02:06/07:02:09/07:44/07:49N/07:58/07:59/07:61	14:02:01
HG01271 (M)	A|A		35:43/35:67/35:79	41:01:00
HG01272 (F)	A|A		35:01:01/35:01:03/35:40N/35:42/35:57/35:94	44:03:01/44:03:03/44:03:04
HG01275 (F)	A|A		07:02:01/07:02:06/07:02:09/07:44/07:49N/07:58/07:59/07:61	35:01:01/35:01:03/35:40N/35:42/35:57/35:94
HG01277 (M)	A|A		51:01:01/51:01:05/51:01:07/51:11N/51:30/51:32/51:48/51:51	57:01:01
HG01341 (M)	A|A		49:01:01	51:01:01/51:01:05/51:01:07/51:11N/51:30/51:32/51:48/51:51
HG01342 (F)	A|A		15:01:01:01/15:01:01:02N/15:01:06/15:01:07/15:102/15:104/15:140/15:146	53:01:01
HG01344 (M)	A|A		35:12:00	51:01:01/51:01:05/51:01:07/51:11N/51:30/51:32/51:48/51:51
HG01345 (F)	A|A		35:08:01	38:01:01
HG01348 (F)	A|A		13:02:01/13:02:05	44:03:01/44:03:03/44:03:04
HG01350 (M)	A|A		40:04:00	57:01:01
HG01351 (F)	A|A		35:01:01/35:01:03/35:40N/35:42/35:57/35:94	49:01:01
HG01353 (M)	A|A		35:43/35:67/35:79	38:01:01
HG01354 (F)	A|A		35:43/35:67/35:79	49:01:01
HG01356 (M)	A|A		18:01:01/18:01:03/18:17N	35:43/35:67/35:79
HG01357 (F)	A|A		27:05:02/27:05:04/27:13	49:01:01
HG01359 (M)	A|A		50:01:00	58:02:00
HG01360 (F)	A|A		42:01:00	44:03:01/44:03:03/44:03:04
HG01365 (M)	A|A		35:10:00	53:01:01
HG01366 (F)	A|A		44:03:01/44:03:03/44:03:04	57:03:01
HG01374 (M)	A|A		35:01:01/35:01:03/35:40N/35:42/35:57/35:94	35:01:01/35:01:03/35:40N/35:42/35:57/35:94
HG01375 (F)	A|A		14:02:01	15:16
HG01377 (M)	A|A		40:02:01/40:56/40:97	51:01:01/51:01:05/51:01:07/51:11N/51:30/51:32/51:48/51:51
HG01378 (F)	A|A		18:01:01/18:01:03/18:17N	35:10:00
HG01383 (M)	A|A		14:02:01	53:01:01
HG01384 (F)	A|A		07:02:01/07:02:06/07:02:09/07:44/07:49N/07:58/07:59/07:61	35:01:01/35:01:03/35:40N/35:42/35:57/35:94
HG01389 (M)	A|A		44:03:01/44:03:03/44:03:04	44:03:01/44:03:03/44:03:04
HG01390 (F)	A|A		14:02:01	39:01:01:01/39:01:01:02L/39:01:03/39:46
HG01437 (M)	A|A		39:06:02	58:01:01/58:11
HG01438 (F)	A|A		07:02:01/07:02:06/07:02:09/07:44/07:49N/07:58/07:59/07:61	07:02:01/07:02:06/07:02:09/07:44/07:49N/07:58/07:59/07:61
HG01440 (M)	A|A		08:01:01/08:19N	39:05:00
HG01441 (F)	A|A		14:02:01	39:05:00
HG01455 (M)	A|A		35:05:00	44:02:01:01/44:02:01:02S/44:19N/44:27/44:66
HG01456 (F)	A|A		35:08:01	40:02:01/40:56/40:97
HG01461 (M)	A|A		40:02:01/40:56/40:97	58:01:01/58:11

**Individual** (1000 Genomes Project Phase 3 Naming)	**rs144012689 Genotype** (minus strand;1000 Genomes)	**Population**	***HLA-B* Allele 1** (from NCBI dbMHC portal)	***HLA-B* Allele 2** (from NCBI dbMHC portal)
HG01462 (F)	A|A		35:08:01	81:01/81:02/81:03
HG01464 (M)	A|A		35:43/35:67/35:79	44:02:01:01/44:02:01:02S/44:19N/44:27/44:66
HG01465 (F)	A|A		51:01:01/51:01:05/51:01:07/51:11N/51:30/51:32/51:48/51:51	51:01:01/51:01:05/51:01:07/51:11N/51:30/51:32/51:48/51:51
HG01488 (M)	A|A		39:11:00	44:03:01/44:03:03/44:03:04
HG01489 (F)	A|A		15:17:01:01/15:17:01:02	35:01:01/35:01:03/35:40N/35:42/35:57/35:94
HG01491 (M)	A|A		14:02:01	57:01:01
HG01492 (F)	A|A		39:06:02	42:02:00
HG01494 (M)	A|A		07:02:01/07:02:06/07:02:09/07:44/07:49N/07:58/07:59/07:61	35:43/35:67/35:79
HG01495 (F)	A|A		18:01:01/18:01:03/18:17N	40:02:01/40:56/40:97
HG01497 (M)	A|A		14:02:01	15:01:01:01/15:01:01:02N/15:01:06/15:01:07/15:102/15:104/15:140/15:146
HG01498 (F)	A|A		35:01:01/35:01:03/35:40N/35:42/35:57/35:94	40:02:01/40:56/40:97
HG01550 (M)	A|A		35:12:00	51:01:01/51:01:05/51:01:07/51:11N/51:30/51:32/51:48/51:51
HG01551 (F)	A|A		07:05:01/07:06	15:16
NA19648 (F)	A|A	1000 Genomes - Mexican Ancestry from Los Angeles-California, USA	07:02:01/07:02:06/07:02:09/07:44/07:49N/07:58/07:59/07:61	51:01:01/51:01:05/51:01:07/51:11N/51:30/51:32/51:48/51:51
NA19649 (M)	A|A		08:01:01/08:19N	39:06:02
NA19651 (F)	A|A		35:01:01/35:01:03/35:40N/35:42/35:57/35:94	40:20:00
NA19652 (M)	A|A		35:08:01	35:01:01/35:01:03/35:40N/35:42/35:57/35:94
NA19654 (F)	A|A		18:06	40:02:01/40:56/40:97
NA19655 (M)	A|A		35:08:01	44:03:01/44:03:03/44:03:04
NA19657 (F)	A|A		08:01:01/08:19N	52:01:02
NA19658 (M)	A|A		44:03:01/44:03:03/44:03:04	55:01:01/55:01:03
NA19661 (M)	A|A		14:02:01	40:02:01/40:56/40:97
NA19663 (F)	A|A		14:02:01	44:05:00
NA19664 (M)	A|A		35:03:01/35:70	39:06:02
NA19669 (F)	A|A		07:02:01/07:02:06/07:02:09/07:44/07:49N/07:58/07:59/07:61	15:03:01/15:103
NA19670 (M)	A|A		14:02:01	38:01:01
NA19676 (M)	A|A		14:02:01	35:01:01/35:01:03/35:40N/35:42/35:57/35:94
NA19678 (F)	A|A		14:01	35:14:01
NA19679 (M)	A|A		14:02:01	57:01:01
NA19681 (F)	A|A		08:01:01/08:19N	39:06:02
NA19682 (M)	A|A		35:17:00	47:01:01:01/47:01:01:02
NA19684 (F)	A|A		07:02:01/07:02:06/07:02:09/07:44/07:49N/07:58/07:59/07:61	35:17:00
NA19716 (F)	A|A		18:01:01/18:01:03/18:17N	27:05:02/27:05:04/27:13
NA19717 (M)	A|A		14:02:01	15:15
NA19719 (F)	A|A		35:03:01/35:70	51:23:00
NA19720 (M)	A|A		15:15	48:01:01/48:09
NA19722 (F)	A|A		18:01:01/18:01:03/18:17N	44:02:01:01/44:02:01:02S/44:19N/44:27/44:66
NA19723 (M)	A|A		15:15	40:02:01/40:56/40:97
NA19725 (F)	A|A		38:01:01	52:01:01/52:07
NA19726 (M)	A|A		15:01:01:01/15:01:01:02N/15:01:06/15:01:07/15:102/15:104/15:140/15:146	39:05:00
NA19728 (F)	A|A		35:12:00	40:05:00
NA19729 (M)	A|A		35:12:00	48:01:01/48:09
NA19731 (F)	A|A		35:17:00	51:02:01
NA19732 (M)	A|A		48:01:01/48:09	51:01:01/51:01:05/51:01:07/51:11N/51:30/51:32/51:48/51:51
NA19746 (F)	A|A		35:17:00	58:02:00
NA19747 (M)	A|A		14:02:01	45:01/45:07
NA19749 (F)	A|A		07:02:01/07:02:06/07:02:09/07:44/07:49N/07:58/07:59/07:61	18:01:01/18:01:03/18:17N
NA19750 (M)	A|A		41:01:00	51:01:01/51:01:05/51:01:07/51:11N/51:30/51:32/51:48/51:51
NA19755 (F)	A|A		35:17:00	51:01:01/51:01:05/51:01:07/51:11N/51:30/51:32/51:48/51:51
NA19756 (M)	A|A		13:02:01/13:02:05	14:01
NA19758 (F)	A|A		35:14:01	48:03:01
NA19759 (M)	A|A		35:05:00	39:06:02
NA19761 (F)	A|A		41:01:00	48:01:01/48:09
NA19762 (M)	A|A		18:01:01/18:01:03/18:17N	35:17:00
NA19770 (F)	A|A		18:01:01/18:01:03/18:17N	51:02:01
NA19771 (M)	A|A		15:16	44:03:01/44:03:03/44:03:04

**Individual** (1000 Genomes Project Phase 3 Naming)	**rs144012689 Genotype** (minus strand;1000 Genomes)	**Population**	***HLA-B* Allele 1** (from NCBI dbMHC portal)	***HLA-B* Allele 2** (from NCBI dbMHC portal)
NA19773 (F)	A|A		14:02:01	41:02:01
NA19774 (M)	A|A		27:05:02/27:05:04/27:13	51:01:01/51:01:05/51:01:07/51:11N/51:30/51:32/51:48/51:51
NA19776 (F)	A|A		07:02:01/07:02:06/07:02:09/07:44/07:49N/07:58/07:59/07:61	35:01:01/35:01:03/35:40N/35:42/35:57/35:94
NA19777 (M)	A|A		15:01:01:01/15:01:01:02N/15:01:06/15:01:07/15:102/15:104/15:140/15:146	35:01:01/35:01:03/35:40N/35:42/35:57/35:94
NA19780 (M)	A|A		40:02:01/40:56/40:97	48:01:01/48:09
NA19782 (F)	A|A		35:01:01/35:01:03/35:40N/35:42/35:57/35:94	42:02:00
NA19783 (M)	A|A		44:03:01/44:03:03/44:03:04	50:01:00
NA19785 (F)	A|A		15:01:01:01/15:01:01:02N/15:01:06/15:01:07/15:102/15:104/15:140/15:146	52:01:02
NA19786 (M)	A|A		18:01:01/18:01:03/18:17N	35:02:01
NA19788 (F)	A|A		45:01/45:07	82:01:00
NA19789 (M)	A|A		18:01:01/18:01:03/18:17N	35:17:00
NA19794 (F)	A|A		51:01:01/51:01:05/51:01:07/51:11N/51:30/51:32/51:48/51:51	56:01/56:24
NA19795 (M)	A|A		35:17:00	48:01:01/48:09
HG00551 (F)	A|A	1000 Genomes - Puerto Rican, Puerto Rico	44:03:01/44:03:03/44:03:04	50:01:00
HG00553 (M)	A|A		44:03:02	58:01:01/58:11
HG00554 (F)	A|A		35:01:01/35:01:03/35:40N/35:42/35:57/35:94	39:01:01:01/39:01:01:02L/39:01:03/39:46
HG00637 (M)	A|A		44:02:01:01/44:02:01:02S/44:19N/44:27/44:66	45:01/45:07
HG00638 (F)	A|A		35:01:01/35:01:03/35:40N/35:42/35:57/35:94	51:01:01/51:01:05/51:01:07/51:11N/51:30/51:32/51:48/51:51
HG00640 (M)	A|A		07:05:01/07:06	18:01:01/18:01:03/18:17N
HG00641 (F)	A|A		07:02:01/07:02:06/07:02:09/07:44/07:49N/07:58/07:59/07:61	15:03:01/15:103
HG00731 (M)	A|A		08:01:01/08:19N	35:02:01
HG00732 (F)	A|A		18:01:01/18:01:03/18:17N	35:01:01/35:01:03/35:40N/35:42/35:57/35:94
HG00734 (F)	A|A		07:02:01/07:02:06/07:02:09/07:44/07:49N/07:58/07:59/07:61	14:02:01
HG00736 (M)	A|A		08:01:01/08:19N	58:01:01/58:11
HG00737 (F)	A|A		35:01:01/35:01:03/35:40N/35:42/35:57/35:94	51:01:01/51:01:05/51:01:07/51:11N/51:30/51:32/51:48/51:51
HG00739 (M)	A|A		35:01:01/35:01:03/35:40N/35:42/35:57/35:94	57:01:01
HG00740 (F)	A|A		27:05:02/27:05:04/27:13	57:03:01
HG01047 (M)	A|A		07:02:01/07:02:06/07:02:09/07:44/07:49N/07:58/07:59/07:61	56:01/56:24
HG01048 (M)	A|A		40:01:01/40:01:02/40:55	44:02:01:01/44:02:01:02S/44:19N/44:27/44:66
HG01049 (F)	A|A		44:03:01/44:03:03/44:03:04	51:01:01/51:01:05/51:01:07/51:11N/51:30/51:32/51:48/51:51
HG01051 (M)	A|A		15:16	35:03:01/35:70
HG01052 (F)	A|A		15:10	52:01:01/52:07
HG01054 (M)	A|A		08:01:01/08:19N	57:03:01
HG01055 (F)	A|A		35:02:01	40:04:00
HG01060 (M)	A|A		35:03:01/35:70	56:01/56:24
HG01061 (F)	A|A		44:03:01/44:03:03/44:03:04	44:03:01/44:03:03/44:03:04
HG01066 (M)	A|A		18:01:01/18:01:03/18:17N	51:01:01/51:01:05/51:01:07/51:11N/51:30/51:32/51:48/51:51
HG01067 (F)	A|A		07:02:01/07:02:06/07:02:09/07:44/07:49N/07:58/07:59/07:61	18:01:01/18:01:03/18:17N
HG01069 (M)	A|A		08:01:01/08:19N	44:02:01:01/44:02:01:02S/44:19N/44:27/44:66
HG01070 (F)	A|A		08:01:01/08:19N	40:01:01/40:01:02/40:55
HG01072 (M)	A|A		14:02:01	50:01:00
HG01073 (F)	A|A		44:03:01/44:03:03/44:03:04	44:03:01/44:03:03/44:03:04
HG01075 (M)	A|A		14:02:01	14:02:01
HG01079 (M)	A|A		39:01:01:01/39:01:01:02L/39:01:03/39:46	40:02:01/40:56/40:97
HG01080 (F)	A|A		15:18	15:03:01/15:103
HG01082 (M)	A|A		39:05:00	44:03:01/44:03:03/44:03:04
HG01083 (F)	A|A		44:02:01:01/44:02:01:02S/44:19N/44:27/44:66	51:01:01/51:01:05/51:01:07/51:11N/51:30/51:32/51:48/51:51
HG01085 (M)	A|A		18:01:01/18:01:03/18:17N	35:11:00
HG01086 (F)	A|A		07:02:01/07:02:06/07:02:09/07:44/07:49N/07:58/07:59/07:61	42:01:00
HG01094 (M)	A|A		44:02:01:01/44:02:01:02S/44:19N/44:27/44:66	52:01:02
HG01095 (F)	A|A		14:02:01	35:11:00
HG01097 (M)	A|A		40:02:01/40:56/40:97	51:01:01/51:01:05/51:01:07/51:11N/51:30/51:32/51:48/51:51
HG01098 (F)	A|A		40:02:01/40:56/40:97	44:03:01/44:03:03/44:03:04
HG01101 (M)	A|A		08:01:01/08:19N	35:01:01/35:01:03/35:40N/35:42/35:57/35:94
HG01102 (F)	A|A		15:16	40:01:01/40:01:02/40:55
HG01104 (M)	A|A		14:02:01	40:02:01/40:56/40:97

**Individual** (1000 Genomes Project Phase 3 Naming)	**rs144012689 Genotype** (minus strand;1000 Genomes)	**Population**	***HLA-B* Allele 1** (from NCBI dbMHC portal)	***HLA-B* Allele 2** (from NCBI dbMHC portal)
HG01105 (F)	A|A		35:01:01/35:01:03/35:40N/35:42/35:57/35:94	49:01:01
HG01107 (M)	A|A		35:03:01/35:70	52:01:02
HG01108 (F)	A|A		35:01:01/35:01:03/35:40N/35:42/35:57/35:94	44:02:01:01/44:02:01:02S/44:19N/44:27/44:66
HG01110 (M)	A|A		35:02:01	45:01/45:07
HG01111 (F)	A|A		14:02:01	35:01:01/35:01:03/35:40N/35:42/35:57/35:94
HG01167 (M)	A|A		45:01/45:07	48:02:00
HG01168 (F)	A|A		18:05	52:01:02
HG01170 (M)	A|A		15:01:01:01/15:01:01:02N/15:01:06/15:01:07/15:102/15:104/15:140/15:146	35:01:01/35:01:03/35:40N/35:42/35:57/35:94
HG01171 (F)	A|A		07:02:01/07:02:06/07:02:09/07:44/07:49N/07:58/07:59/07:61	56:01/56:24
HG01173 (M)	A|A		07:02:01/07:02:06/07:02:09/07:44/07:49N/07:58/07:59/07:61	07:02:01/07:02:06/07:02:09/07:44/07:49N/07:58/07:59/07:61
HG01174 (F)	A|A		35:12:00	53:01:01
HG01176 (M)	A|A		07:05:01/07:06	44:03:01/44:03:03/44:03:04
HG01177 (F)	A|A		51:01:01/51:01:05/51:01:07/51:11N/51:30/51:32/51:48/51:51	53:01:01
HG01182 (M)	A|A		35:08:01	44:03:01/44:03:03/44:03:04
HG01183 (F)	A|A		35:03:01/35:70	78:01:00
HG01187 (M)	A|A		44:02:01:01/44:02:01:02S/44:19N/44:27/44:66	57:01:01
HG01188 (F)	A|A		35:02:01	44:03:01/44:03:03/44:03:04
HG01190 (M)	A|A		15:20	18:01:01/18:01:03/18:17N
HG01191 (F)	A|A		07:02:01/07:02:06/07:02:09/07:44/07:49N/07:58/07:59/07:61	39:05:00
HG01197 (M)	A|A		07:02:01/07:02:06/07:02:09/07:44/07:49N/07:58/07:59/07:61	56:01/56:24
HG01198 (F)	A|A		40:01:01/40:01:02/40:55	44:03:01/44:03:03/44:03:04
HG01204 (M)	A|A		15:01:01:01/15:01:01:02N/15:01:06/15:01:07/15:102/15:104/15:140/15:146	44:03:01/44:03:03/44:03:04
HG01205 (F)	A|A		07:02:01/07:02:06/07:02:09/07:44/07:49N/07:58/07:59/07:61	35:01:01/35:01:03/35:40N/35:42/35:57/35:94
HG01241 (M)	A|A		07:02:01/07:02:06/07:02:09/07:44/07:49N/07:58/07:59/07:61	15:03:01/15:103
HG01242 (F)	A|A		35:01:01/35:01:03/35:40N/35:42/35:57/35:94	44:03:01/44:03:03/44:03:04
HG01247 (M)	A|A		52:01:01/52:07	57:01:01
HG01248 (F)	A|A		14:02:01	51:01:01/51:01:05/51:01:07/51:11N/51:30/51:32/51:48/51:51
NA06985 (F)	A|A	rn and Western European from Utah, USA	7:02:01	57:01:00
NA06986 (M)	A|A		44:03:01/44:03:03/44:03:04	44:03:01/44:03:03/44:03:04
NA06994 (M)	A|A		40:02:00	8:01
NA07000 (F)	A|A		44:02:00	40:01:00
NA07037 (F)	A|A		15:10	40:01:00
NA07048 (M)	A|A		44:02:01:01	7:02
NA07051 (M)	A|A		15:01/15:12/15:19	7:02
NA07056 (F)	A|A		8:01	57:01:00
NA07347 (M)	A|A		18:01	44:02:00
NA07357 (M)	A|A		8:01	39:06:00
NA10847 (F)	A|A		8:01	18:01
NA10851 (M)	A|A		40:01:00	8:01
NA11829 (M)	A|A		15:01/15:28	44:02:00
NA11830 (F)	A|A		14:01	14:02
NA11831 (M)	A|A		8:01	7:02
NA11832 (F)	A|A		40:02:00	27:03/27:51/27:52/27:09
NA11840 (F)	A|A		27:03:00	57:01:00
NA11843 (M)	A|A		15:01	51:01:00
NA11881 (M)	A|A		7:02	7:02
NA11892 (F)	A|A		13:02:01/13:02:05	49:01:00
NA11893 (M)	A|A		15:01/15:28	44:02:00
NA11894 (F)	A|A		8:01	55:01:01/55:01:03
NA11918 (F)	A|A		7:02	44:02:00
NA11919 (M)	A|A		15:01	57:01:00
NA11920 (F)	A|A		44:02:00	55:01:01/55:01:03/55:02
NA11930 (M)	A|A		13:02:01/13:02:05	13:02:01/13:02:05
NA11931 (F)	A|A		44:02:00	7:02
NA11992 (M)	A|A		35:01:00	8:01
NA11994 (M)	A|A		7:02	51:01:00

**Individual** (1000 Genomes Project Phase 3 Naming)	**rs144012689 Genotype** (minus strand;1000 Genomes)	**Population**	***HLA-B* Allele 1** (from NCBI dbMHC portal)	***HLA-B* Allele 2** (from NCBI dbMHC portal)
NA11995 (F)	A|A	1000 Genomes - Northe	57:01:00	8:01
NA12003 (M)	A|A		8:01	13:02:01/13:02:05
NA12004 (F)	A|A		7:02	7:02
NA12005 (M)	A|A		7:02	27:03/27:52/27:09
NA12006 (F)	A|A		18:01	15:01/15:12/15:19
NA12043 (M)	A|A		35:01:00	38:01:01
NA12044 (F)	A|A		7:02	7:02
NA12045 (M)	A|A		35:01:00	44:02:00
NA12046 (F)	A|A		57:01:00	50:01:01
NA12058 (F)	A|A		40:01:00	40:01:00
NA12144 (M)	A|A		35:01/35:07	44:02:00
NA12154 (M)	A|A		8:01	40:01:00
NA12155 (M)	A|A		8:01	44:02:00
NA12156 (F)	A|A		51:01:00	50:01:01
NA12234 (F)	A|A		44:03:01/44:03:03/44:03:04	40:02:00
NA12249 (F)	A|A		40:01:00	44
NA12272 (M)	A|A		44:02:00	44:02:00
NA12273 (F)	A|A		35:01/35:07	44:02:00
NA12275 (F)	A|A		14:01	7:02
NA12282 (M)	A|A		8:01	37:01:00
NA12283 (F)	A|A		15:01/15:12/15:19	57:01:00
NA12286 (M)	A|A		57:01:00	13:02:01/13:02:05
NA12287 (F)	A|A		15:01/15:03	15:01/15:26/15:12/15:19
HG00171 (F)	A|A		13:02:01/13:02:05	51:01:01/51:01:05/51:01:07/51:11N/51:30/51:32/51:48/51:51
HG00173 (F)	A|A		40:01:01/40:01:02/40:55	44:02:01:01/44:02:01:02S/44:19N/44:27/44:66
HG00174 (F)	A|A		15:01:01:01/15:01:01:02N/15:01:06/15:01:07/15:102/15:104/15:140/15:146	38:01:01
HG00176 (F)	A|A		15:01:01:01/15:01:01:02N/15:01:06/15:01:07/15:102/15:104/15:140/15:146	35:01:01/35:01:03/35:40N/35:42/35:57/35:94
HG00177 (F)	A|A		13:02:01/13:02:05	40:01:01/40:01:02/40:55
HG00178 (F)	A|A		07:02:01/07:02:06/07:02:09/07:44/07:49N/07:58/07:59/07:61	08:01:01/08:19N
HG00179 (F)	A|A		07:02:01/07:02:06/07:02:09/07:44/07:49N/07:58/07:59/07:61	35:01:01/35:01:03/35:40N/35:42/35:57/35:94
HG00180 (F)	A|A		40:02:01/40:56/40:97	41:01:00
HG00181 (M)	A|A		15:01:01:01/15:01:01:02N/15:01:06/15:01:07/15:102/15:104/15:140/15:146	55:01:01/55:01:03
HG00182 (M)	A|A		35:01:01/35:01:03/35:40N/35:42/35:57/35:94	44:02:01:01/44:02:01:02S/44:19N/44:27/44:66
HG00183 (M)	A|A		40:02:01/40:56/40:97	55:01:01/55:01:03
HG00185 (M)	A|A		40:02:01/40:56/40:97	44:02:01:01/44:02:01:02S/44:19N/44:27/44:66
HG00186 (M)	A|A		15:18	27:05:02/27:05:04/27:13
HG00187 (M)	A|A		15:01:01:01/15:01:01:02N/15:01:06/15:01:07/15:102/15:104/15:140/15:146	55:01:01/55:01:03
HG00188 (M)	A|A		39:01:01:01/39:01:01:02L/39:01:03/39:46	44:03:01/44:03:03/44:03:04
HG00189 (M)	A|A		07:02:01/07:02:06/07:02:09/07:44/07:49N/07:58/07:59/07:61	08:01:01/08:19N
HG00190 (M)	A|A		35:01:01/35:01:03/35:40N/35:42/35:57/35:94	35:01:01/35:01:03/35:40N/35:42/35:57/35:94
HG00266 (F)	A|A		08:01:01/08:19N	35:01:01/35:01:03/35:40N/35:42/35:57/35:94
HG00267 (M)	A|A		41:01:00	57:01:01
HG00268 (F)	A|A		07:02:01/07:02:06/07:02:09/07:44/07:49N/07:58/07:59/07:61	18:01:01/18:01:03/18:17N
HG00269 (F)	A|A		07:02:01/07:02:06/07:02:09/07:44/07:49N/07:58/07:59/07:61	35:01:01/35:01:03/35:40N/35:42/35:57/35:94
HG00271 (M)	A|A		27:05:02/27:05:04/27:13	47:01:01:01/47:01:01:02
HG00272 (F)	A|A		08:01:01/08:19N	15:01:01:01/15:01:01:02N/15:01:06/15:01:07/15:102/15:104/15:140/15:146
HG00273 (M)	A|A		07:02:01/07:02:06/07:02:09/07:44/07:49N/07:58/07:59/07:61	35:01:01/35:01:03/35:40N/35:42/35:57/35:94
HG00274 (F)	A|A		15:01:01:01/15:01:01:02N/15:01:06/15:01:07/15:102/15:104/15:140/15:146	35:01:01/35:01:03/35:40N/35:42/35:57/35:94
HG00275 (F)	A|A		07:02:01/07:02:06/07:02:09/07:44/07:49N/07:58/07:59/07:61	15:01:01:01/15:01:01:02N/15:01:06/15:01:07/15:102/15:104/15:140/15:146
HG00276 (F)	A|A		35:01:01/35:01:03/35:40N/35:42/35:57/35:94	51:01:01/51:01:05/51:01:07/51:11N/51:30/51:32/51:48/51:51
HG00277 (M)	A|A		07:02:01/07:02:06/07:02:09/07:44/07:49N/07:58/07:59/07:61	56:01/56:24
HG00278 (M)	A|A		39:01:01:01/39:01:01:02L/39:01:03/39:46	51:01:01/51:01:05/51:01:07/51:11N/51:30/51:32/51:48/51:51
HG00280 (M)	A|A		08:01:01/08:19N	27:05:02/27:05:04/27:13
HG00281 (F)	A|A		15:01:01:01/15:01:01:02N/15:01:06/15:01:07/15:102/15:104/15:140/15:146	35:01:01/35:01:03/35:40N/35:42/35:57/35:94
HG00282 (F)	A|A		07:02:01/07:02:06/07:02:09/07:44/07:49N/07:58/07:59/07:61	40:01:01/40:01:02/40:55
HG00284 (M)	A|A		08:01:01/08:19N	57:01:01

**Individual** (1000 Genomes Project Phase 3 Naming)	**rs144012689 Genotype** (minus strand;1000 Genomes)	**Population**	***HLA-B* Allele 1** (from NCBI dbMHC portal)	***HLA-B* Allele 2** (from NCBI dbMHC portal)
HG00285 (F)	A|A	1000 Genomes - Finnish, Finland	15:01:01:01/15:01:01:02N/15:01:06/15:01:07/15:102/15:104/15:140/15:146	40:01:01/40:01:02/40:55
HG00306 (F)	A|A		08:01:01/08:19N	27:05:02/27:05:04/27:13
HG00308 (M)	A|A		35:01:01/35:01:03/35:40N/35:42/35:57/35:94	35:01:01/35:01:03/35:40N/35:42/35:57/35:94
HG00309 (F)	A|A		27:05:02/27:05:04/27:13	35:01:01/35:01:03/35:40N/35:42/35:57/35:94
HG00310 (M)	A|A		07:02:01/07:02:06/07:02:09/07:44/07:49N/07:58/07:59/07:61	15:01:01:01/15:01:01:02N/15:01:06/15:01:07/15:102/15:104/15:140/15:146
HG00311 (M)	A|A		07:02:01/07:02:06/07:02:09/07:44/07:49N/07:58/07:59/07:61	44:02:01:01/44:02:01:02S/44:19N/44:27/44:66
HG00313 (F)	A|A		07:02:01/07:02:06/07:02:09/07:44/07:49N/07:58/07:59/07:61	35:01:01/35:01:03/35:40N/35:42/35:57/35:94
HG00315 (F)	A|A		13:02:01/13:02:05	56:01/56:24
HG00318 (F)	A|A		07:02:01/07:02:06/07:02:09/07:44/07:49N/07:58/07:59/07:61	13:02:01/13:02:05
HG00319 (F)	A|A		07:02:01/07:02:06/07:02:09/07:44/07:49N/07:58/07:59/07:61	56:01/56:24
HG00320 (F)	A|A		15:01:01:01/15:01:01:02N/15:01:06/15:01:07/15:102/15:104/15:140/15:146	35:01:01/35:01:03/35:40N/35:42/35:57/35:94
HG00321 (M)	A|A		15:01:01:01/15:01:01:02N/15:01:06/15:01:07/15:102/15:104/15:140/15:146	40:01:01/40:01:02/40:55
HG00323 (F)	A|A		18:01:01/18:01:03/18:17N	40:01:01/40:01:02/40:55
HG00324 (F)	A|A		35:01:01/35:01:03/35:40N/35:42/35:57/35:94	40:01:01/40:01:02/40:55
HG00325 (M)	A|A		35:01:01/35:01:03/35:40N/35:42/35:57/35:94	35:01:01/35:01:03/35:40N/35:42/35:57/35:94
HG00326 (F)	A|A		40:01:01/40:01:02/40:55	57:01:01
HG00327 (F)	A|A		08:01:01/08:19N	44:02:01:01/44:02:01:02S/44:19N/44:27/44:66
HG00328 (F)	A|A		15:01:01:01/15:01:01:02N/15:01:06/15:01:07/15:102/15:104/15:140/15:146	27:05:02/27:05:04/27:13
HG00329 (M)	A|A		15:01:01:01/15:01:01:02N/15:01:06/15:01:07/15:102/15:104/15:140/15:146	51:01:01/51:01:05/51:01:07/51:11N/51:30/51:32/51:48/51:51
HG00330 (F)	A|A		15:01:01:01/15:01:01:02N/15:01:06/15:01:07/15:102/15:104/15:140/15:146	57:01:01
HG00331 (F)	A|A		15:01:01:01/15:01:01:02N/15:01:06/15:01:07/15:102/15:104/15:140/15:146	35:01:01/35:01:03/35:40N/35:42/35:57/35:94
HG00332 (F)	A|A		07:02:01/07:02:06/07:02:09/07:44/07:49N/07:58/07:59/07:61	35:01:01/35:01:03/35:40N/35:42/35:57/35:94
HG00334 (F)	A|A		15:01:01:01/15:01:01:02N/15:01:06/15:01:07/15:102/15:104/15:140/15:146	39:01:01:01/39:01:01:02L/39:01:03/39:46
HG00335 (M)	A|A		15:01:01:01/15:01:01:02N/15:01:06/15:01:07/15:102/15:104/15:140/15:146	27:05:02/27:05:04/27:13
HG00336 (M)	A|A		08:01:01/08:19N	35:01:01/35:01:03/35:40N/35:42/35:57/35:94
HG00337 (F)	A|A		07:02:01/07:02:06/07:02:09/07:44/07:49N/07:58/07:59/07:61	07:02:01/07:02:06/07:02:09/07:44/07:49N/07:58/07:59/07:61
HG00338 (M)	A|A		35:01:01/35:01:03/35:40N/35:42/35:57/35:94	44:02:01:01/44:02:01:02S/44:19N/44:27/44:66
HG00339 (F)	A|A		27:05:02/27:05:04/27:13	40:02:01/40:56/40:97
HG00341 (M)	A|A		40:01:01/40:01:02/40:55	57:01:01
HG00342 (M)	A|A		13:02:01/13:02:05	44:02:01:01/44:02:01:02S/44:19N/44:27/44:66
HG00343 (F)	A|A		35:01:01/35:01:03/35:40N/35:42/35:57/35:94	39:01:01:01/39:01:01:02L/39:01:03/39:46
HG00344 (F)	A|A		07:02:01/07:02:06/07:02:09/07:44/07:49N/07:58/07:59/07:61	07:02:01/07:02:06/07:02:09/07:44/07:49N/07:58/07:59/07:61
HG00345 (M)	A|A		08:01:01/08:19N	35:01:01/35:01:03/35:40N/35:42/35:57/35:94
HG00346 (F)	A|A		18:01:01/18:01:03/18:17N	51:01:01/51:01:05/51:01:07/51:11N/51:30/51:32/51:48/51:51
HG00349 (F)	A|A		08:01:01/08:19N	27:05:02/27:05:04/27:13
HG00350 (F)	A|A		15:01:01:01/15:01:01:02N/15:01:06/15:01:07/15:102/15:104/15:140/15:146	15:01:01:01/15:01:01:02N/15:01:06/15:01:07/15:102/15:104/15:140/15:146
HG00351 (M)	A|A		07:02:01/07:02:06/07:02:09/07:44/07:49N/07:58/07:59/07:61	38:01:01
HG00353 (F)	A|A		18:01:01/18:01:03/18:17N	44:02:01:01/44:02:01:02S/44:19N/44:27/44:66
HG00355 (F)	A|A		44:03:01/44:03:03/44:03:04	47:01:01:01/47:01:01:02
HG00356 (F)	A|A		08:01:01/08:19N	40:01:01/40:01:02/40:55
HG00357 (F)	A|A		08:01:01/08:19N	57:01:01
HG00358 (M)	A|A		27:05:02/27:05:04/27:13	47:01:01:01/47:01:01:02
HG00360 (M)	A|A		39:01:01:01/39:01:01:02L/39:01:03/39:46	44:02:01:01/44:02:01:02S/44:19N/44:27/44:66
HG00361 (F)	A|A		07:02:01/07:02:06/07:02:09/07:44/07:49N/07:58/07:59/07:61	35:01:01/35:01:03/35:40N/35:42/35:57/35:94
HG00362 (F)	A|A		27:05:02/27:05:04/27:13	44:02:01:01/44:02:01:02S/44:19N/44:27/44:66
HG00364 (F)	A|A		35:01:01/35:01:03/35:40N/35:42/35:57/35:94	67:01:02
HG00365 (F)	A|A		44:02:01:01/44:02:01:02S/44:19N/44:27/44:66	56:01/56:24
HG00366 (M)	A|A		07:02:01/07:02:06/07:02:09/07:44/07:49N/07:58/07:59/07:61	15:01:01:01/15:01:01:02N/15:01:06/15:01:07/15:102/15:104/15:140/15:146
HG00367 (F)	A|A		35:01:01/35:01:03/35:40N/35:42/35:57/35:94	37:01:01
HG00368 (F)	A|A		13:02:01/13:02:05	56:01/56:24
HG00369 (M)	A|A		27:05:02/27:05:04/27:13	44:02:01:01/44:02:01:02S/44:19N/44:27/44:66
HG00371 (M)	A|A		35:01:01/35:01:03/35:40N/35:42/35:57/35:94	40:01:01/40:01:02/40:55
HG00372 (M)	A|A		27:05:02/27:05:04/27:13	40:01:01/40:01:02/40:55
HG00373 (F)	A|A		15:01:01:01/15:01:01:02N/15:01:06/15:01:07/15:102/15:104/15:140/15:146	56:01/56:24
HG00375 (M)	A|A		13:02:01/13:02:05	40:01:01/40:01:02/40:55
HG00376 (F)	A|A		40:01:01/40:01:02/40:55	51:01:01/51:01:05/51:01:07/51:11N/51:30/51:32/51:48/51:51

**Individual** (1000 Genomes Project Phase 3 Naming)	**rs144012689 Genotype** (minus strand;1000 Genomes)	**Population**	***HLA-B* Allele 1** (from NCBI dbMHC portal)	***HLA-B* Allele 2** (from NCBI dbMHC portal)
HG00378 (F)	A|A		35:01:01/35:01:03/35:40N/35:42/35:57/35:94	40:01:01/40:01:02/40:55
HG00379 (F)	A|A		07:02:01/07:02:06/07:02:09/07:44/07:49N/07:58/07:59/07:61	44:02:01:01/44:02:01:02S/44:19N/44:27/44:66
HG00380 (F)	A|A		07:02:01/07:02:06/07:02:09/07:44/07:49N/07:58/07:59/07:61	08:01:01/08:19N
HG00381 (F)	A|A		15:01:01:01/15:01:01:02N/15:01:06/15:01:07/15:102/15:104/15:140/15:146	39:01:01:01/39:01:01:02L/39:01:03/39:46
HG00382 (M)	A|A		07:02:01/07:02:06/07:02:09/07:44/07:49N/07:58/07:59/07:61	44:02:01:01/44:02:01:02S/44:19N/44:27/44:66
HG00383 (F)	A|A		18:01:01/18:01:03/18:17N	27:05:02/27:05:04/27:13
HG00384 (F)	A|A		35:01:01/35:01:03/35:40N/35:42/35:57/35:94	40:02:01/40:56/40:97
HG00096 (M)	A|A	mes - British from England and Scotland, UK	08:01:01/08:19N	44:03:01/44:03:03/44:03:04
HG00097 (F)	A|A		07:02:01/07:02:06/07:02:09/07:44/07:49N/07:58/07:59/07:61	07:02:01/07:02:06/07:02:09/07:44/07:49N/07:58/07:59/07:61
HG00099 (F)	A|A		08:01:01/08:19N	44:02:01:01/44:02:01:02S/44:19N/44:27/44:66
HG00100 (F)	A|A		08:01:01/08:19N	57:01:01
HG00101 (M)	A|A		27:05:02/27:05:04/27:13	57:01:01
HG00102 (F)	A|A		07:02:01/07:02:06/07:02:09/07:44/07:49N/07:58/07:59/07:61	08:01:01/08:19N
HG00103 (M)	A|A		35:01:01/35:01:03/35:40N/35:42/35:57/35:94	40:01:01/40:01:02/40:55
HG00105 (M)	A|A		07:02:01/07:02:06/07:02:09/07:44/07:49N/07:58/07:59/07:61	44:03:01/44:03:03/44:03:04
HG00106 (F)	A|A		08:01:01/08:19N	35:03:01/35:70
HG00107 (M)	A|A		07:02:01/07:02:06/07:02:09/07:44/07:49N/07:58/07:59/07:61	40:01:01/40:01:02/40:55
HG00108 (M)	A|A		35:02:01	40:01:01/40:01:02/40:55
HG00109 (M)	A|A		08:01:01/08:19N	44:02:01:01/44:02:01:02S/44:19N/44:27/44:66
HG00110 (F)	A|A		44:02:01:01/44:02:01:02S/44:19N/44:27/44:66	55:01:01/55:01:03
HG00111 (F)	A|A		27:05:02/27:05:04/27:13	57:01:01
HG00112 (M)	A|A		14:02:01	51:01:01/51:01:05/51:01:07/51:11N/51:30/51:32/51:48/51:51
HG00113 (M)	A|A		07:02:01/07:02:06/07:02:09/07:44/07:49N/07:58/07:59/07:61	44:03:01/44:03:03/44:03:04
HG00114 (M)	A|A		07:02:01/07:02:06/07:02:09/07:44/07:49N/07:58/07:59/07:61	08:01:01/08:19N
HG00115 (M)	A|A		27:05:02/27:05:04/27:13	27:05:02/27:05:04/27:13
HG00116 (M)	A|A		07:02:01/07:02:06/07:02:09/07:44/07:49N/07:58/07:59/07:61	14:02:01
HG00117 (M)	A|A		08:01:01/08:19N	55:01:01/55:01:03
HG00118 (F)	A|A		07:02:01/07:02:06/07:02:09/07:44/07:49N/07:58/07:59/07:61	40:01:01/40:01:02/40:55
HG00119 (M)	A|A		35:03:01/35:70	38:01:01
HG00120 (F)	A|A		08:01:01/08:19N	27:05:02/27:05:04/27:13
HG00121 (F)	A|A		07:02:01/07:02:06/07:02:09/07:44/07:49N/07:58/07:59/07:61	40:01:01/40:01:02/40:55
HG00122 (F)	A|A		14:01	57:01:01
HG00123 (F)	A|A		44:03:01/44:03:03/44:03:04	57:01:01
HG00125 (F)	A|A		14:02:01	44:02:01:01/44:02:01:02S/44:19N/44:27/44:66
HG00126 (M)	A|A		35:03:01/35:70	44:03:01/44:03:03/44:03:04
HG00127 (F)	A|A		38:01:01	45:01/45:07
HG00128 (F)	A|A		14:02:01	27:05:02/27:05:04/27:13
HG00129 (M)	A|A		13:02:01/13:02:05	35:01:01/35:01:03/35:40N/35:42/35:57/35:94
HG00130 (F)	A|A		15:03:01/15:103	44:03:01/44:03:03/44:03:04
HG00131 (M)	A|A		37:01:01	57:01:01
HG00132 (F)	A|A		35:03:01/35:70	44:02:01:01/44:02:01:02S/44:19N/44:27/44:66
HG00133 (F)	A|A		44:02:01:01/44:02:01:02S/44:19N/44:27/44:66	53:01:01
HG00136 (M)	A|A		44:02:01:01/44:02:01:02S/44:19N/44:27/44:66	51:01:01/51:01:05/51:01:07/51:11N/51:30/51:32/51:48/51:51
HG00137 (F)	A|A		08:01:01/08:19N	44:02:01:01/44:02:01:02S/44:19N/44:27/44:66
HG00138 (M)	A|A		35:01:01/35:01:03/35:40N/35:42/35:57/35:94	51:01:01/51:01:05/51:01:07/51:11N/51:30/51:32/51:48/51:51
HG00139 (M)	A|A		44:03:01/44:03:03/44:03:04	44:03:01/44:03:03/44:03:04
HG00140 (M)	A|A		40:01:01/40:01:02/40:55	57:01:01
HG00141 (M)	A|A		15:01:01:01/15:01:01:02N/15:01:06/15:01:07/15:102/15:104/15:140/15:146	35:01:01/35:01:03/35:40N/35:42/35:57/35:94
HG00142 (M)	A|A		45:01/45:07	57:01:01
HG00143 (M)	A|A		07:02:01/07:02:06/07:02:09/07:44/07:49N/07:58/07:59/07:61	57:01:01
HG00145 (M)	A|A		07:02:01/07:02:06/07:02:09/07:44/07:49N/07:58/07:59/07:61	40:01:01/40:01:02/40:55
HG00146 (F)	A|A		08:01:01/08:19N	35:01:01/35:01:03/35:40N/35:42/35:57/35:94
HG00148 (M)	A|A		40:01:01/40:01:02/40:55	44:02:01:01/44:02:01:02S/44:19N/44:27/44:66
HG00149 (M)	A|A		13:02:01/13:02:05	44:03:01/44:03:03/44:03:04
HG00150 (F)	A|A		27:05:02/27:05:04/27:13	51:01:01/51:01:05/51:01:07/51:11N/51:30/51:32/51:48/51:51
HG00151 (M)	A|A		08:01:01/08:19N	55:01:01/55:01:03

**Individual** (1000 Genomes Project Phase 3 Naming)	**rs144012689 Genotype** (minus strand;1000 Genomes)	**Population**	***HLA-B* Allele 1** (from NCBI dbMHC portal)	***HLA-B* Allele 2** (from NCBI dbMHC portal)
HG00154 (F)	A|A	1000 Genom	35:01:01/35:01:03/35:40N/35:42/35:57/35:94	39:01:01:01/39:01:01:02L/39:01:03/39:46
HG00155 (M)	A|A		14:01	57:01:01
HG00157 (M)	A|A		07:02:01/07:02:06/07:02:09/07:44/07:49N/07:58/07:59/07:61	45:01/45:07
HG00158 (F)	A|A		07:02:01/07:02:06/07:02:09/07:44/07:49N/07:58/07:59/07:61	56:01/56:24
HG00159 (M)	A|A		07:02:01/07:02:06/07:02:09/07:44/07:49N/07:58/07:59/07:61	55:01:01/55:01:03
HG00160 (M)	A|A		13:02:01/13:02:05	39:06:02
HG00231 (F)	A|A		44:02:01:01/44:02:01:02S/44:19N/44:27/44:66	51:01:01/51:01:05/51:01:07/51:11N/51:30/51:32/51:48/51:51
HG00232 (F)	A|A		07:02:01/07:02:06/07:02:09/07:44/07:49N/07:58/07:59/07:61	27:05:02/27:05:04/27:13
HG00233 (F)	A|A		35:01:01/35:01:03/35:40N/35:42/35:57/35:94	44:02:01:01/44:02:01:02S/44:19N/44:27/44:66
HG00234 (M)	A|A		18:01:01/18:01:03/18:17N	44:02:01:01/44:02:01:02S/44:19N/44:27/44:66
HG00235 (F)	A|A		07:02:01/07:02:06/07:02:09/07:44/07:49N/07:58/07:59/07:61	44:02:01:01/44:02:01:02S/44:19N/44:27/44:66
HG00236 (F)	A|A		07:02:01/07:02:06/07:02:09/07:44/07:49N/07:58/07:59/07:61	39:06:02
HG00237 (F)	A|A		35:01:01/35:01:03/35:40N/35:42/35:57/35:94	39:06:02
HG00238 (F)	A|A		51:01:01/51:01:05/51:01:07/51:11N/51:30/51:32/51:48/51:51	56:01/56:24
HG00239 (F)	A|A		37:01:01	44:02:01:01/44:02:01:02S/44:19N/44:27/44:66
HG00240 (F)	A|A		51:01:01/51:01:05/51:01:07/51:11N/51:30/51:32/51:48/51:51	56:01/56:24
HG00242 (M)	A|A		27:05:02/27:05:04/27:13	27:05:02/27:05:04/27:13
HG00243 (M)	A|A		44:03:01/44:03:03/44:03:04	44:02:01:01/44:02:01:02S/44:19N/44:27/44:66
HG00244 (M)	A|A		15:01:01:01/15:01:01:02N/15:01:06/15:01:07/15:102/15:104/15:140/15:146	58:01:01/58:11
HG00245 (F)	A|A		40:01:01/40:01:02/40:55	40:01:01/40:01:02/40:55
HG00246 (M)	A|A		27:05:02/27:05:04/27:13	51:01:01/51:01:05/51:01:07/51:11N/51:30/51:32/51:48/51:51
HG00250 (F)	A|A		40:01:01/40:01:02/40:55	57:01:01
HG00251 (M)	A|A		08:01:01/08:19N	39:01:01:01/39:01:01:02L/39:01:03/39:46
HG00252 (M)	A|A		40:02:01/40:56/40:97	44:03:01/44:03:03/44:03:04
HG00253 (F)	A|A		07:02:01/07:02:06/07:02:09/07:44/07:49N/07:58/07:59/07:61	58:01:01/58:11
HG00254 (F)	A|A		14:02:01	14:02:01
HG00255 (F)	A|A		39:01:01:01/39:01:01:02L/39:01:03/39:46	44:03:01/44:03:03/44:03:04
HG00256 (M)	A|A		14:01	15:01:01:01/15:01:01:02N/15:01:06/15:01:07/15:102/15:104/15:140/15:146
HG00257 (F)	A|A		08:01:01/08:19N	40:02:01/40:56/40:97
HG00258 (F)	A|A		40:01:01/40:01:02/40:55	40:01:01/40:01:02/40:55
HG00259 (F)	A|A		13:02:01/13:02:05	40:01:01/40:01:02/40:55
HG00260 (M)	A|A		18:01:01/18:01:03/18:17N	44:02:01:01/44:02:01:02S/44:19N/44:27/44:66
HG00261 (F)	A|A		55:01:01/55:01:03	57:01:01
HG00262 (F)	A|A		44:03:01/44:03:03/44:03:04	44:02:01:01/44:02:01:02S/44:19N/44:27/44:66
HG00263 (F)	A|A		07:02:01/07:02:06/07:02:09/07:44/07:49N/07:58/07:59/07:61	41:01:00
HG00264 (M)	A|A		27:05:02/27:05:04/27:13	44:02:01:01/44:02:01:02S/44:19N/44:27/44:66
HG00265 (M)	A|A		08:01:01/08:19N	08:01:01/08:19N
HG01334 (M)	A|A		44:02:01:01/44:02:01:02S/44:19N/44:27/44:66	44:02:01:01/44:02:01:02S/44:19N/44:27/44:66
NA20502 (F)	A|A		07:02:01/07:02:06/07:02:09/07:44/07:49N/07:58/07:59/07:61	35:02:01
NA20504 (F)	A|A		55:01:01/55:01:03	57:01:01
NA20505 (F)	A|A		35:03:01/35:70	57:01:01
NA20506 (F)	A|A		08:01:01/08:19N	18:01:01/18:01:03/18:17N
NA20508 (F)	A|A		41:02:01	57:01:01
NA20509 (M)	A|A		13:02:01/13:02:05	51:01:01/51:01:05/51:01:07/51:11N/51:30/51:32/51:48/51:51
NA20510 (M)	A|A		15:17:01:01/15:17:01:02	55:01:01/55:01:03
NA20512 (M)	A|A		27:05:02/27:05:04/27:13	51:01:01/51:01:05/51:01:07/51:11N/51:30/51:32/51:48/51:51
NA20515 (M)	A|A		07:02:01/07:02:06/07:02:09/07:44/07:49N/07:58/07:59/07:61	18:01:01/18:01:03/18:17N
NA20516 (M)	A|A		35:08:01	44:02:01:01/44:02:01:02S/44:19N/44:27/44:66
NA20517 (F)	A|A		35:02:01	44:03:01/44:03:03/44:03:04
NA20518 (M)	A|A		07:02:01/07:02:06/07:02:09/07:44/07:49N/07:58/07:59/07:61	35:01:01/35:01:03/35:40N/35:42/35:57/35:94
NA20519 (M)	A|A		13:02:01/13:02:05	51:01:01/51:01:05/51:01:07/51:11N/51:30/51:32/51:48/51:51
NA20520 (M)	A|A		18:01:01/18:01:03/18:17N	35:01:01/35:01:03/35:40N/35:42/35:57/35:94
NA20521 (M)	A|A		14:01	49:01:01
NA20522 (F)	A|A		35:03:01/35:70	39:01:01:01/39:01:01:02L/39:01:03/39:46
NA20524 (M)	A|A		44:03:01/44:03:03/44:03:04	49:01:01
NA20525 (M)	A|A		08:01:01/08:19N	51:01:01/51:01:05/51:01:07/51:11N/51:30/51:32/51:48/51:51

**Individual** (1000 Genomes Project Phase 3 Naming)	**rs144012689 Genotype** (minus strand;1000 Genomes)	**Population**	***HLA-B* Allele 1** (from NCBI dbMHC portal)	***HLA-B* Allele 2** (from NCBI dbMHC portal)
NA20527 (M)	A|A	1000 Genomes - Italian from Tuscany, Italy	07:02:01/07:02:06/07:02:09/07:44/07:49N/07:58/07:59/07:61	07:05:01/07:06
NA20528 (M)	A|A		41:02:01	44:03:01/44:03:03/44:03:04
NA20529 (F)	A|A		15:01:01:01/15:01:01:02N/15:01:06/15:01:07/15:102/15:104/15:140/15:146	55:01:01/55:01:03
NA20530 (F)	A|A		07:02:01/07:02:06/07:02:09/07:44/07:49N/07:58/07:59/07:61	13:02:01/13:02:05
NA20531 (F)	A|A		35:01:01/35:01:03/35:40N/35:42/35:57/35:94	44:02:01:01/44:02:01:02S/44:19N/44:27/44:66
NA20534 (M)	A|A		15:01:01:01/15:01:01:02N/15:01:06/15:01:07/15:102/15:104/15:140/15:146	27:05:02/27:05:04/27:13
NA20535 (F)	A|A		07:02:01/07:02:06/07:02:09/07:44/07:49N/07:58/07:59/07:61	51:01:01/51:01:05/51:01:07/51:11N/51:30/51:32/51:48/51:51
NA20538 (M)	A|A		44:02:01:01/44:02:01:02S/44:19N/44:27/44:66	51:01:01/51:01:05/51:01:07/51:11N/51:30/51:32/51:48/51:51
NA20539 (M)	A|A		07:02:01/07:02:06/07:02:09/07:44/07:49N/07:58/07:59/07:61	58:01:01/58:11
NA20540 (F)	A|A		44:02:01:01/44:02:01:02S/44:19N/44:27/44:66	51:01:01/51:01:05/51:01:07/51:11N/51:30/51:32/51:48/51:51
NA20541 (F)	A|A		08:01:01/08:19N	35:03:01/35:70
NA20542 (F)	A|A		07:02:01/07:02:06/07:02:09/07:44/07:49N/07:58/07:59/07:61	14:02:01
NA20543 (M)	A|A		08:01:01/08:19N	53:01:01
NA20544 (M)	A|A		14:02:01	44:02:01:01/44:02:01:02S/44:19N/44:27/44:66
NA20581 (M)	A|A		51:01:01/51:01:05/51:01:07/51:11N/51:30/51:32/51:48/51:51	57:01:01
NA20582 (F)	A|A		38:01:01	55:01:01/55:01:03
NA20585 (F)	A|A		07:02:01/07:02:06/07:02:09/07:44/07:49N/07:58/07:59/07:61	07:02:01/07:02:06/07:02:09/07:44/07:49N/07:58/07:59/07:61
NA20586 (M)	A|A		08:01:01/08:19N	18:01:01/18:01:03/18:17N
NA20588 (M)	A|A		08:01:01/08:19N	51:01:01/51:01:05/51:01:07/51:11N/51:30/51:32/51:48/51:51
NA20589 (F)	A|A		18:01:01/18:01:03/18:17N	35:03:01/35:70
NA20752 (M)	A|A		13:02:01/13:02:05	51:01:01/51:01:05/51:01:07/51:11N/51:30/51:32/51:48/51:51
NA20753 (F)	A|A		13:02:01/13:02:05	35:02:01
NA20754 (M)	A|A		35:01:01/35:01:03/35:40N/35:42/35:57/35:94	58:01:01/58:11
NA20755 (M)	A|A		41:02:01	44:03:01/44:03:03/44:03:04
NA20756 (F)	A|A		49:01:01	51:01:01/51:01:05/51:01:07/51:11N/51:30/51:32/51:48/51:51
NA20757 (F)	A|A		18:01:01/18:01:03/18:17N	38:01:01
NA20758 (M)	A|A		08:01:01/08:19N	55:01:01/55:01:03
NA20759 (M)	A|A		18:01:01/18:01:03/18:17N	44:03:01/44:03:03/44:03:04
NA20760 (F)	A|A		15:01:01:01/15:01:01:02N/15:01:06/15:01:07/15:102/15:104/15:140/15:146	57:01:01
NA20761 (F)	A|A		15:17:01:01/15:17:01:02	51:01:01/51:01:05/51:01:07/51:11N/51:30/51:32/51:48/51:51
NA20765 (M)	A|A		55:01:01/55:01:03	58:01:01/58:11
NA20766 (F)	A|A		35:02:01	51:01:01/51:01:05/51:01:07/51:11N/51:30/51:32/51:48/51:51
NA20768 (F)	A|A		18:01:01/18:01:03/18:17N	44:03:01/44:03:03/44:03:04
NA20769 (F)	A|A		15:01:01:01/15:01:01:02N/15:01:06/15:01:07/15:102/15:104/15:140/15:146	58:01:01/58:11
NA20770 (M)	A|A		50:01:00	51:01:01/51:01:05/51:01:07/51:11N/51:30/51:32/51:48/51:51
NA20771 (F)	A|A		13:02:01/13:02:05	57:01:01
NA20772 (F)	A|A		40:01:01/40:01:02/40:55	51:01:01/51:01:05/51:01:07/51:11N/51:30/51:32/51:48/51:51
NA20773 (F)	A|A		49:01:01	49:01:01
NA20774 (F)	A|A		50:01:00	51:01:01/51:01:05/51:01:07/51:11N/51:30/51:32/51:48/51:51
NA20775 (F)	A|A		35:01:01/35:01:03/35:40N/35:42/35:57/35:94	50:01:00
NA20778 (M)	A|A		07:02:01/07:02:06/07:02:09/07:44/07:49N/07:58/07:59/07:61	35:03:01/35:70
NA20783 (M)	A|A		13:02:01/13:02:05	15:17:01:01/15:17:01:02
NA20785 (M)	A|A		18:01:01/18:01:03/18:17N	52:01:01/52:07
NA20786 (F)	A|A		51:01:01/51:01:05/51:01:07/51:11N/51:30/51:32/51:48/51:51	51:01:01/51:01:05/51:01:07/51:11N/51:30/51:32/51:48/51:51
NA20787 (M)	A|A		38:01:01	39:06:02
NA20790 (F)	A|A		07:02:01/07:02:06/07:02:09/07:44/07:49N/07:58/07:59/07:61	37:01:01
NA20792 (M)	A|A		15:09	35:01:01/35:01:03/35:40N/35:42/35:57/35:94
NA20795 (F)	A|A		18:01:01/18:01:03/18:17N	39:06:02
NA20796 (M)	A|A		07:02:01/07:02:06/07:02:09/07:44/07:49N/07:58/07:59/07:61	51:01:01/51:01:05/51:01:07/51:11N/51:30/51:32/51:48/51:51
NA20797 (F)	A|A		07:05:01/07:06	14:02:01
NA20798 (M)	A|A		07:02:01/07:02:06/07:02:09/07:44/07:49N/07:58/07:59/07:61	08:01:01/08:19N
NA20799 (F)	A|A		15:18	57:02:00
NA20800 (F)	A|A		37:01:01	44:03:01/44:03:03/44:03:04
NA20801 (M)	A|A		18:01:01/18:01:03/18:17N	44:02:01:01/44:02:01:02S/44:19N/44:27/44:66
NA20802 (F)	A|A		08:01:01/08:19N	40:01:01/40:01:02/40:55
NA20803 (M)	A|A		44:03:01/44:03:03/44:03:04	53:01:01

**Individual** (1000 Genomes Project Phase 3 Naming)	**rs144012689 Genotype** (minus strand;1000 Genomes)	**Population**	***HLA-B* Allele 1** (from NCBI dbMHC portal)	***HLA-B* Allele 2** (from NCBI dbMHC portal)
NA20804 (F)	A|A		35:02:01	51:01:01/51:01:05/51:01:07/51:11N/51:30/51:32/51:48/51:51
NA20805 (M)	A|A		18:01:01/18:01:03/18:17N	44:02:01:01/44:02:01:02S/44:19N/44:27/44:66
NA20806 (M)	A|A		35:03:01/35:70	51:01:01/51:01:05/51:01:07/51:11N/51:30/51:32/51:48/51:51
NA20807 (F)	A|A		07:02:01/07:02:06/07:02:09/07:44/07:49N/07:58/07:59/07:61	08:01:01/08:19N
NA20808 (F)	A|A		07:05:01/07:06	51:01:01/51:01:05/51:01:07/51:11N/51:30/51:32/51:48/51:51
NA20809 (M)	A|A		07:02:01/07:02:06/07:02:09/07:44/07:49N/07:58/07:59/07:61	44:03:01/44:03:03/44:03:04
NA20810 (M)	A|A		35:03:01/35:70	39:06:02
NA20811 (M)	A|A		35:08:01	57:01:01
NA20812 (M)	A|A		18:01:01/18:01:03/18:17N	41:01:00
NA20813 (F)	A|A		07:02:01/07:02:06/07:02:09/07:44/07:49N/07:58/07:59/07:61	44:02:01:01/44:02:01:02S/44:19N/44:27/44:66
NA20814 (M)	A|A		35:01:01/35:01:03/35:40N/35:42/35:57/35:94	44:02:01:01/44:02:01:02S/44:19N/44:27/44:66
NA20815 (M)	A|A		15:01:01:01/15:01:01:02N/15:01:06/15:01:07/15:102/15:104/15:140/15:146	44:02:01:01/44:02:01:02S/44:19N/44:27/44:66
NA20818 (F)	A|A		35:02:01	35:01:01/35:01:03/35:40N/35:42/35:57/35:94
NA20819 (F)	A|A		35:01:01/35:01:03/35:40N/35:42/35:57/35:94	51:01:01/51:01:05/51:01:07/51:11N/51:30/51:32/51:48/51:51
NA20826 (F)	A|A		13:02:01/13:02:05	38:01:01
NA20828 (F)	A|A		49:01:01	51:01:01/51:01:05/51:01:07/51:11N/51:30/51:32/51:48/51:51
NA18526 (F)	A|A		40:01:00	58:01:00
NA18530 (M)	A|A		44:03:01/44:03:03/44:03:04	48:01:01/48:09
NA18532 (F)	A|A		13:02:01/13:02:05	15:18
NA18534 (M)	A|A		15:01:01:01/15:01:01:02N/15:01:06/15:01:07/15:102/15:104/15:140/15:146	58:01:01/58:11
NA18536 (M)	A|A		48:01:01/48:09	51:02:01
NA18537 (F)	A|A		38:02:00	46:01:00
NA18542 (F)	A|A		46:01:00	58:01:00
NA18543 (M)	A|A		13:01:01	46:01:01/46:15N
NA18544 (M)	A|A		07:02:01/07:02:06/07:02:09/07:44/07:49N/07:58/07:59/07:61	52:01:01/52:07
NA18545 (F)	A|A		40:01:00	40:01:00
NA18546 (M)	A|A		40:01:01/40:01:02/40:55	58:01:01/58:11
NA18547 (F)	A|T		15:02	38:01:00
NA18548 (M)	A|A		40:02:01/40:56/40:97	51:01:01/51:01:05/51:01:07/51:11N/51:30/51:32/51:48/51:51
NA18549 (M)	A|A		55:02:01	58:01:01/58:11
NA18550 (F)	A|A		13:01	46:01:00
NA18552 (F)	A|A		1.668055556	1.917361111
NA18555 (F)	A|A		7:05	54:01:00
NA18557 (M)	A|A		35:03:01/35:70	44:02:01:01/44:02:01:02S/44:19N/44:27/44:66
NA18558 (M)	A|A		48:01:00	67:01:00
NA18559 (M)	T|A		15:02	37:01:01
NA18561 (M)	A|A		39:01:00	59:01:00
NA18562 (M)	A|A		51:01:00	52:01:00
NA18563 (M)	A|A		48:01:00	51:01:00
NA18564 (F)	A|A		13:01	13:02:01/13:02:05
NA18566 (F)	A|A		35:01:00	8:01
NA18570 (F)	A|A		15:01	15:11
NA18571 (F)	A|A		40:01:00	51:01:00
NA18572 (M)	A|A		15:01	54:01:00
NA18573 (F)	A|A		46:01:00	54:01:00
NA18577 (F)	A|A		15:01:01:01/15:01:01:02N/15:01:06/15:01:07/15:102/15:104/15:140/15:146	40:01:01/40:01:02/40:55
NA18579 (F)	A|A		52:01:01/52:07	81:01/81:02/81:03
NA18582 (F)	A|A		40:01:00	52:01:00
NA18592 (F)	A|A		15:18	40:06:00
NA18593 (F)	A|A		37:01:00	40:01:00
NA18595 (F)	A|A		40:01:01/40:01:02/40:55	44:02:01:01/44:02:01:02S/44:19N/44:27/44:66
NA18596 (F)	A|A		35:01:01/35:01:03/35:40N/35:42/35:57/35:94	46:01:01/46:15N
NA18597 (F)	A|A		35:01:01/35:01:03/35:40N/35:42/35:57/35:94	51:01:01/51:01:05/51:01:07/51:11N/51:30/51:32/51:48/51:51
NA18599 (F)	A|A		13:02:01/13:02:05	54:01/54:17
NA18602 (F)	A|A		15:07	54:01/54:17
NA18603 (M)	A|A		13:02:01/13:02:05	40:06:00

**Individual** (1000 Genomes Project Phase 3 Naming)	**rs144012689 Genotype** (minus strand;1000 Genomes)	**Population**	***HLA-B* Allele 1** (from NCBI dbMHC portal)	***HLA-B* Allele 2** (from NCBI dbMHC portal)
NA18605 (M)	A|A	mes - Han from Beijing, China + Japanese from Tokyo, Japan	15:01	38:02:00
NA18606 (M)	A|A		13:01:01	46:01:01/46:15N
NA18608 (M)	A|A		48:01:00	51:01:00
NA18609 (M)	A|A		35:03:00	38:02:00
NA18610 (F)	A|A		35:01:01/35:01:03/35:40N/35:42/35:57/35:94	52:01:01/52:07
NA18611 (M)	A|A		46:01:00	51:01:00
NA18612 (M)	A|A		40:06:01:01/40:06:01:02	57:01:01
NA18613 (M)	A|A		13:01:01	51:01:01/51:01:05/51:01:07/51:11N/51:30/51:32/51:48/51:51
NA18614 (F)	A|A		46:01:01/46:15N	58:01:01/58:11
NA18615 (F)	A|A		13:02:01/13:02:05	46:01:01/46:15N
NA18616 (F)	A|A		15:18	40:01:01/40:01:02/40:55
NA18617 (F)	A|A		40:01:01/40:01:02/40:55	58:01:01/58:11
NA18618 (F)	A|A		15:27:01	40:01:01/40:01:02/40:55
NA18619 (F)	A|A		40:01:01/40:01:02/40:55	58:01:01/58:11
NA18620 (M)	A|A		40:01:00	48:01:00
NA18621 (M)	A|A		15:01	15:18
NA18622 (M)	A|A		35:03:00	51:01:00
NA18623 (M)	A|A		13:02:01/13:02:05	40:01:00
NA18624 (M)	A|A		13:02:01/13:02:05	52:01:00
NA18625 (F)	A|A		37:01:01	46:01:01/46:15N
NA18626 (F)	A|A		27:07:00	50:01:00
NA18627 (F)	A|A		15:01:01:01/15:01:01:02N/15:01:06/15:01:07/15:102/15:104/15:140/15:146	51:01:01/51:01:05/51:01:07/51:11N/51:30/51:32/51:48/51:51
NA18628 (F)	A|A		46:01:01/46:15N	58:01:01/58:11
NA18630 (F)	A|T		15:02	40:01:01/40:01:02/40:55
NA18631 (F)	A|A		35:01:01/35:01:03/35:40N/35:42/35:57/35:94	40:01:01/40:01:02/40:55
NA18632 (M)	A|A		46:01:00	58:01:00
NA18633 (M)	A|A		54:01:00	54:01:00
NA18634 (F)	A|A		40:02:01/40:56/40:97	46:01:01/46:15N
NA18635 (M)	A|A		40:06:00	51:01:00
NA18636 (M)	A|A		40:01:00	46:01:00
NA18637 (M)	A|A		15:01	67:01:00
NA18638 (M)	A|A		40:01:01/40:01:02/40:55	58:01:01/58:11
NA18639 (M)	A|T		15:02	54:01/54:17
NA18640 (F)	A|A		46:01:01/46:15N	46:01:01/46:15N
NA18641 (F)	A|A		40:01:01/40:01:02/40:55	51:01:01/51:01:05/51:01:07/51:11N/51:30/51:32/51:48/51:51
NA18642 (F)	A|A		07:02:01/07:02:06/07:02:09/07:44/07:49N/07:58/07:59/07:61	38:01:01
NA18643 (M)	A|A		40:06:01:01/40:06:01:02	46:01:01/46:15N
NA18644 (F)	A|A		40:02:01/40:56/40:97	58:01:01/58:11
NA18647 (M)	A|A		13:02:01/13:02:05	44:02:01:01/44:02:01:02S/44:19N/44:27/44:66
NA18740 (M)	A|A		13:01:01	40:01:01/40:01:02/40:55
NA18745 (M)	A|A		38:02:01/38:18	48:01:01/48:09
NA18747 (M)	A|A		15:27:01	35:03:01/35:70
NA18748 (M)	A|A		07:02:01/07:02:06/07:02:09/07:44/07:49N/07:58/07:59/07:61	58:01:01/58:11
NA18749 (M)	A|A		13:02:01/13:02:05	44:03:02
NA18757 (M)	A|A		07:02:01/07:02:06/07:02:09/07:44/07:49N/07:58/07:59/07:61	55:02:01
NA18939 (F)	A|A		27:04:01	67:01:01
NA18940 (M)	A|A		46:01:00	52:01:00
NA18941 (F)	A|A		15:07	40:01:01/40:01:02/40:55
NA18942 (F)	A|A		35:01:00	44:03:01/44:03:03/44:03:04
NA18943 (M)	A|A		35:01:00	46:01:00
NA18944 (M)	A|A		40:02:00	51:01:00
NA18945 (M)	A|A		15:01	44:03:01/44:03:03/44:03:04
NA18946 (F)	A|A		07:02:01/07:02:06/07:02:09/07:44/07:49N/07:58/07:59/07:61	52:01:01/52:07
NA18947 (F)	A|A		52:01:00	52:01:00
NA18948 (M)	A|A		7:02	13:02:01/13:02:05
NA18949 (F)	A|A		07:02:01/07:02:06/07:02:09/07:44/07:49N/07:58/07:59/07:61	40:02:01/40:56/40:97

**Individual** (1000 Genomes Project Phase 3 Naming)	**rs144012689 Genotype** (minus strand;1000 Genomes)	**Population**	***HLA-B*** **Allele 1** (from NCBI dbMHC portal)	***HLA-B*** **Allele 2** (from NCBI dbMHC portal)
NA18951 (F)	A|A	1000 Genom	40:01:00	59:01:00
NA18952 (M)	A|A		7:02	51:01:00
NA18953 (M)	A|A		40:01:00	40:02:00
NA18954 (F)	A|A		13:01:01	52:01:01/52:07
NA18956 (F)	A|A		15:01	40:02:00
NA18957 (F)	A|A		52:01:01/52:07	52:01:01/52:07
NA18959 (M)	A|A		52:01:00	67:01:00
NA18960 (M)	A|A		40:06:00	54:01:00
NA18961 (M)	A|A		51:01:00	54:01:00
NA18962 (M)	A|A		07:02:01/07:02:06/07:02:09/07:44/07:49N/07:58/07:59/07:61	52:01:01/52:07
NA18963 (F)	A|A		40:06:01:01/40:06:01:02	44:03:01/44:03:03/44:03:04
NA18964 (F)	A|A		35:01:00	40:02:00
NA18965 (M)	A|A		40:06:00	56:01:00
NA18966 (M)	A|A		15:01	35:01:00
NA18967 (M)	A|A		40:02:00	51:01:00
NA18968 (F)	A|A		40:01:01/40:01:02/40:55	51:01:01/51:01:05/51:01:07/51:11N/51:30/51:32/51:48/51:51
NA18969 (F)	A|A		48:01:00	52:01:00
NA18970 (M)	A|A		15:18	54:01:00
NA18971 (M)	A|A		40:02:00	40:02:00
NA18972 (F)	A|A		40:02:00	59:01:00
NA18973 (F)	A|A		15:07	46:01:00
NA18974 (M)	A|A		39:01:00	40:01:00
NA18975 (F)	A|A		7:02	15:01
NA18976 (F)	A|A		44:03:01/44:03:03/44:03:04	44:03:01/44:03:03/44:03:04
NA18977 (M)	A|A		35:01:01/35:01:03/35:40N/35:42/35:57/35:94	55:02:01
NA18978 (F)	A|A		40:02:00	44:02:00
NA18979 (F)	A|A		52:01:01/52:07	55:02:01
NA18980 (F)	A|A		13:01	40:01:00
NA18981 (F)	A|A		51:01:00	51:01:00
NA18987 (F)	A|A		40:06:00	46:01:00
NA18990 (M)	A|A		40:02:00	44:03:01/44:03:03/44:03:04
NA18991 (F)	A|A		40:02:00	52:01:00
NA18992 (F)	A|A		44:03:01/44:03:03/44:03:04	52:01:00
NA18993 (F)	A|A		35:01:01/35:01:03/35:40N/35:42/35:57/35:94	39:01:01:01/39:01:01:02L/39:01:03/39:46
NA18994 (M)	A|A		7:02	40:02:00
NA18995 (M)	A|A		15:07	44:03:01/44:03:03/44:03:04
NA18997 (F)	A|A		15:01:01:01/15:01:01:02N/15:01:06/15:01:07/15:102/15:104/15:140/15:146	39:01:01:01/39:01:01:02L/39:01:03/39:46
NA18998 (F)	A|A		52:01:00	54:01:00
NA18999 (F)	A|A		40:06:00	46:01:00
NA19000 (M)	A|A		54:01:00	67:01:00
NA19001 (F)	A|A		07:02:01/07:02:06/07:02:09/07:44/07:49N/07:58/07:59/07:61	52:01:01/52:07
NA19002 (F)	A|A		37:01:01	44:03:01/44:03:03/44:03:04
NA19003 (F)	A|A		39:01:00	39:01:00
NA19005 (M)	A|A		58:01:00	67:01:00
NA19007 (M)	A|A		35:01:00	46:01:00
NA19009 (M)	A|A		40:01:01/40:01:02/40:55	52:01:01/52:07
NA19010 (F)	A|A		35:01:01/35:01:03/35:40N/35:42/35:57/35:94	35:01:01/35:01:03/35:40N/35:42/35:57/35:94
NA19012 (M)	A|A		35:01:01/35:01:03/35:40N/35:42/35:57/35:94	35:01:01/35:01:03/35:40N/35:42/35:57/35:94
NA19054 (F)	A|A		35:01:01/35:01:03/35:40N/35:42/35:57/35:94	44:03:01/44:03:03/44:03:04
NA19055 (M)	A|A		07:02:01/07:02:06/07:02:09/07:44/07:49N/07:58/07:59/07:61	59:01:00
NA19056 (M)	A|A		40:06:01:01/40:06:01:02	40:02:01/40:56/40:97
NA19057 (F)	A|A		40:06:01:01/40:06:01:02	59:01:00
NA19058 (M)	A|A		39:02:01	54:01/54:17
NA19059 (F)	A|A		46:01:01/46:15N	52:01:01/52:07
NA19060 (M)	A|A		40:02:01/40:56/40:97	51:01:01/51:01:05/51:01:07/51:11N/51:30/51:32/51:48/51:51
NA19062 (M)	A|A		07:02:01/07:02:06/07:02:09/07:44/07:49N/07:58/07:59/07:61	44:03:01/44:03:03/44:03:04

**Individual** (1000 Genomes Project Phase 3 Naming)	**rs144012689 Genotype** (minus strand;1000 Genomes)	**Population**	***HLA-B* Allele 1** (from NCBI dbMHC portal)	***HLA-B* Allele 2** (from NCBI dbMHC portal)
NA19063 (M)	A|A		15:18	15:01:01:01/15:01:01:02N/15:01:06/15:01:07/15:102/15:104/15:140/15:146
NA19064 (F)	A|A		15:18	46:01:01/46:15N
NA19065 (F)	A|A		39:01:01:01/39:01:01:02L/39:01:03/39:46	51:01:01/51:01:05/51:01:07/51:11N/51:30/51:32/51:48/51:51
NA19066 (M)	A|A		51:01:01/51:01:05/51:01:07/51:11N/51:30/51:32/51:48/51:51	52:01:01/52:07
NA19067 (M)	A|A		15:18	54:01/54:17
NA19068 (M)	A|A		40:02:01/40:56/40:97	51:01:01/51:01:05/51:01:07/51:11N/51:30/51:32/51:48/51:51
NA19070 (M)	A|A		46:01:01/46:15N	52:01:01/52:07
NA19072 (M)	A|A		46:01:01/46:15N	58:01:01/58:11
NA19074 (F)	A|A		40:02:01/40:56/40:97	52:01:01/52:07
NA19075 (M)	A|A		15:01:01:01/15:01:01:02N/15:01:06/15:01:07/15:102/15:104/15:140/15:146	56:01/56:24
NA19076 (M)	A|A		40:02:01/40:56/40:97	40:02:01/40:56/40:97
NA19077 (F)	A|A		37:01:01	51:01:01/51:01:05/51:01:07/51:11N/51:30/51:32/51:48/51:51
NA19078 (F)	A|A		40:06:01:01/40:06:01:02	54:01/54:17
NA19079 (M)	A|A		44:03:01/44:03:03/44:03:04	48:01:01/48:09
NA19080 (F)	A|A		48:01:01/48:09	58:01:01/58:11
NA19081 (F)	A|A		35:01:01/35:01:03/35:40N/35:42/35:57/35:94	52:01:01/52:07
NA19082 (M)	A|A		40:02:01/40:56/40:97	51:01:01/51:01:05/51:01:07/51:11N/51:30/51:32/51:48/51:51
NA19083 (M)	A|A		15:01:01:01/15:01:01:02N/15:01:06/15:01:07/15:102/15:104/15:140/15:146	44:03:01/44:03:03/44:03:04
NA19084 (F)	A|A		40:01:01/40:01:02/40:55	40:01:01/40:01:02/40:55
NA19085 (M)	A|A		44:03:01/44:03:03/44:03:04	52:01:01/52:07
NA19086 (M)	A|A		40:06:01:01/40:06:01:02	51:01:01/51:01:05/51:01:07/51:11N/51:30/51:32/51:48/51:51
NA19087 (F)	A|A		35:01:01/35:01:03/35:40N/35:42/35:57/35:94	44:03:01/44:03:03/44:03:04
NA19088 (M)	A|A		15:07	15:01:01:01/15:01:01:02N/15:01:06/15:01:07/15:102/15:104/15:140/15:146
HG00403 (M)	A|A		13:01:01	35:01:01/35:01:03/35:40N/35:42/35:57/35:94
HG00404 (F)	A|A		15:12/15:19	27:04:01
HG00406 (M)	A|T		07:02:01/07:02:06/07:02:09/07:44/07:49N/07:58/07:59/07:61	15:02
HG00407 (F)	A|A		40:01:01/40:01:02/40:55	40:02:01/40:56/40:97
HG00419 (F)	A|A		13:01:01	40:01:01/40:01:02/40:55
HG00421 (M)	A|A		13:02:01/13:02:05	46:01:01/46:15N
HG00422 (F)	A|A		13:01:01	44:03:01/44:03:03/44:03:04
HG00428 (F)	A|A		13:02:01/13:02:05	46:01:01/46:15N
HG00436 (M)	A|A		48:01:01/48:09	54:01/54:17
HG00437 (F)	A|A		40:01:01/40:01:02/40:55	51:01:01/51:01:05/51:01:07/51:11N/51:30/51:32/51:48/51:51
HG00442 (M)	A|A		40:01:01/40:01:02/40:55	40:02:01/40:56/40:97
HG00443 (F)	A|A		13:01:01	54:01/54:17
HG00445 (M)	A|A		07:02:01/07:02:06/07:02:09/07:44/07:49N/07:58/07:59/07:61	67:01:01
HG00446 (F)	A|A		13:01:01	40:01:01/40:01:02/40:55
HG00448 (M)	A|A		46:01:01/46:15N	56:01/56:24
HG00449 (F)	A|A		39:01:01:01/39:01:01:02L/39:01:03/39:46	46:01:01/46:15N
HG00451 (M)	A|A		15:01:01:01/15:01:01:02N/15:01:06/15:01:07/15:102/15:104/15:140/15:146	40:01:01/40:01:02/40:55
HG00452 (F)	A|A		40:01:01/40:01:02/40:55	55:02:01
HG00457 (M)	A|A		39:01:01:01/39:01:01:02L/39:01:03/39:46	46:01:01/46:15N
HG00458 (F)	A|A		15:01:01:01/15:01:01:02N/15:01:06/15:01:07/15:102/15:104/15:140/15:146	46:01:01/46:15N
HG00463 (M)	A|T		15:02	46:01:01/46:15N
HG00464 (F)	A|A		15:01:01:01/15:01:01:02N/15:01:06/15:01:07/15:102/15:104/15:140/15:146	40:01:01/40:01:02/40:55
HG00472 (M)	A|A		15:01:01:01/15:01:01:02N/15:01:06/15:01:07/15:102/15:104/15:140/15:146	57:01:01
HG00473 (F)	A|A		46:01:01/46:15N	58:01:01/58:11
HG00475 (M)	A|A		13:01:01	58:01:01/58:11
HG00476 (F)	A|A		40:01:01/40:01:02/40:55	40:01:01/40:01:02/40:55
HG00478 (M)	A|A		40:01:01/40:01:02/40:55	40:01:01/40:01:02/40:55
HG00479 (F)	A|A		46:01:01/46:15N	55:02:01
HG00500 (M)	A|A		40:01:01/40:01:02/40:55	46:01:01/46:15N
HG00513 (F)	A|A		40:01:01/40:01:02/40:55	55:02:01
HG00524 (M)	A|A		40:06:01:01/40:06:01:02	46:01:01/46:15N
HG00525 (F)	A|A		07:02:01/07:02:06/07:02:09/07:44/07:49N/07:58/07:59/07:61	35:01:01/35:01:03/35:40N/35:42/35:57/35:94
HG00530 (M)	A|A		39:01:01:01/39:01:01:02L/39:01:03/39:46	39:01:01:01/39:01:01:02L/39:01:03/39:46

**Individual** (1000 Genomes Project Phase 3 Naming)	**rs144012689 Genotype** (minus strand;1000 Genomes)	**Population**	***HLA-B* Allele 1** (from NCBI dbMHC portal)	***HLA-B* Allele 2** (from NCBI dbMHC portal)
HG00531 (F)	A|A	1000 Genomes - Han from south, China	35:01:01/35:01:03/35:40N/35:42/35:57/35:94	46:01:01/46:15N
HG00533 (M)	A|A		39:01:01:01/39:01:01:02L/39:01:03/39:46	48:01:01/48:09
HG00534 (F)	A|A		46:01:01/46:15N	57:01:01
HG00536 (M)	A|T		15:02	46:01:01/46:15N
HG00537 (F)	A|A		46:01:01/46:15N	46:01:01/46:15N
HG00542 (M)	A|A		46:01:01/46:15N	58:01:01/58:11
HG00543 (F)	A|A		35:01:01/35:01:03/35:40N/35:42/35:57/35:94	46:01:01/46:15N
HG00556 (M)	A|A		40:01:01/40:01:02/40:55	40:01:01/40:01:02/40:55
HG00557 (F)	A|A		37:01:01	48:01:01/48:09
HG00559 (M)	A|A		55:02:01	58:01:01/58:11
HG00560 (F)	A|T		15:02	39:01:01:01/39:01:01:02L/39:01:03/39:46
HG00565 (M)	A|A		40:01:01/40:01:02/40:55	40:01:01/40:01:02/40:55
HG00566 (F)	A|A		46:01:01/46:15N	58:01:01/58:11
HG00580 (M)	A|A		13:02:01/13:02:05	51:01:01/51:01:05/51:01:07/51:11N/51:30/51:32/51:48/51:51
HG00581 (F)	A|A		15:11:01	40:01:01/40:01:02/40:55
HG00583 (M)	A|A		40:01:01/40:01:02/40:55	55:02:01
HG00584 (F)	A|A		40:01:01/40:01:02/40:55	40:01:01/40:01:02/40:55
HG00589 (M)	A|A		13:01:01	51:01:01/51:01:05/51:01:07/51:11N/51:30/51:32/51:48/51:51
HG00590 (F)	A|A		15:25:01	35:01:01/35:01:03/35:40N/35:42/35:57/35:94
HG00592 (M)	A|A		08:01:01/08:19N	51:01:01/51:01:05/51:01:07/51:11N/51:30/51:32/51:48/51:51
HG00593 (F)	A|A		27:04:01	35:01:01/35:01:03/35:40N/35:42/35:57/35:94
HG00595 (M)	A|T		15:02	40:01:01/40:01:02/40:55
HG00596 (F)	A|A		15:01:01:01/15:01:01:02N/15:01:06/15:01:07/15:102/15:104/15:140/15:146	40:01:01/40:01:02/40:55
HG00607 (M)	A|A		40:01:01/40:01:02/40:55	58:01:01/58:11
HG00608 (F)	A|A		40:01:01/40:01:02/40:55	40:02:01/40:56/40:97
HG00610 (M)	A|A		40:06:01:01/40:06:01:02	51:01:01/51:01:05/51:01:07/51:11N/51:30/51:32/51:48/51:51
HG00611 (F)	A|A		46:01:01/46:15N	46:01:01/46:15N
HG00613 (M)	A|A		40:01:01/40:01:02/40:55	46:01:01/46:15N
HG00614 (F)	A|A		13:01:01	55:02:01
HG00619 (M)	A|A		40:01:01/40:01:02/40:55	56:01/56:24
HG00620 (F)	T|A		15:02	51:01:01/51:01:05/51:01:07/51:11N/51:30/51:32/51:48/51:51
HG00625 (M)	A|A		15:01:01:01/15:01:01:02N/15:01:06/15:01:07/15:102/15:104/15:140/15:146	35:01:01/35:01:03/35:40N/35:42/35:57/35:94
HG00626 (F)	A|A		15:27:01	40:01:01/40:01:02/40:55
HG00628 (M)	A|A		13:01:01	38:02:01/38:18
HG00629 (F)	A|A		35:01:01/35:01:03/35:40N/35:42/35:57/35:94	40:02:01/40:56/40:97
HG00634 (M)	A|A		39:01:01:01/39:01:01:02L/39:01:03/39:46	39:01:01:01/39:01:01:02L/39:01:03/39:46
HG00650 (M)	A|A		46:01:01/46:15N	58:01:01/58:11
HG00651 (F)	A|A		13:01:01	46:01:01/46:15N
HG00653 (M)	A|A		51:01:01/51:01:05/51:01:07/51:11N/51:30/51:32/51:48/51:51	58:01:01/58:11
HG00654 (F)	A|A		40:01:01/40:01:02/40:55	40:01:01/40:01:02/40:55
HG00656 (M)	A|A		39:01:01:01/39:01:01:02L/39:01:03/39:46	46:01:01/46:15N
HG00657 (F)	A|A		46:01:01/46:15N	51:01:02
HG00662 (M)	A|A		40:01:01/40:01:02/40:55	46:01:01/46:15N
HG00663 (F)	A|A		40:01:01/40:01:02/40:55	51:01:02
HG00671 (M)	T|A		15:02	48:03:01
HG00672 (F)	A|A		38:02:01/38:18	51:02:01
HG00683 (M)	A|A		40:01:01/40:01:02/40:55	46:01:01/46:15N
HG00684 (F)	A|A		13:01:01	13:01:01
HG00689 (M)	A|A		40:01:01/40:01:02/40:55	48:01:01/48:09
HG00690 (F)	A|A		39:01:01:01/39:01:01:02L/39:01:03/39:46	40:01:01/40:01:02/40:55
HG00692 (M)	A|A		15:01:01:01/15:01:01:02N/15:01:06/15:01:07/15:102/15:104/15:140/15:146	46:01:01/46:15N
HG00693 (F)	A|A		13:01:01	40:01:01/40:01:02/40:55
HG00698 (M)	A|A		38:02:01/38:18	58:01:01/58:11
HG00699 (F)	A|A		52:01:01/52:07	58:01:01/58:11
HG00701 (M)	T|A		15:02	46:01:01/46:15N
HG00704 (M)	A|A		13:01:01	38:02:01/38:18

**Individual** (1000 Genomes Project Phase 3 Naming)	**rs144012689 Genotype** (minus strand;1000 Genomes)	**Population**	***HLA-B* Allele 1** (from NCBI dbMHC portal)	***HLA-B* Allele 2** (from NCBI dbMHC portal)
HG00705 (F)	A|A		27:04:01	54:01/54:17
HG00707 (M)	A|A		40:01:01/40:01:02/40:55	46:01:01/46:15N
HG00708 (F)	A|A		46:01:01/46:15N	57:01:01
