# Supplementary material for: A Screening Test for HLA-B∗15:02 in a Large United States Patient Cohort Identifies Broader Risk of Carbamazepine-Induced Adverse Events
Source: Front Pharmacol. 2019 Mar 26;10:149. doi: 10.3389/fphar.2019.00149 (PMC6443844; doi:10.3389/fphar.2019.00149)
Supplement: Supplementary file 4 [file Table_2.pdf]

**Supplementary Table S2. *HLA-B* and rs144012689 genotypes of 32 *HLA-B*15:02* positive control samples**

| Sample ID | ***HLA-B*** **Allele 1** | ***HLA-B*** **Allele 2** | **rs14401289 PCR genotype** |
|---|---|---|---|
| NA17019 | 15:02 | 15:11 | T/A |
| NA23093 | 15:02 | 15:02 | T/T |
| NA23090 | 15:02 | 51:01 | T/A |
| NA18547 | 15:02 | 38:01 | T/A |
| HG00406 | 15:02 | 07:02:01/07:02:06/07:02:09/07:44/07:49N/07:58/07:59/07:61 | T/A |
| HG00536 | 15:02 | 46:01:01/46:15N | T/A |
| HG00560 | 15:02 | 39:01:01:01/39:01:01:02L/39:01:03/39:46 | T/A |
| HG00595 | 15:02 | 40:01:01/40:01:02/40:55 | T/A |
| HG00620 | 15:02 | 51:01:01/51:01:05/51:01:07/51:11N/51:30/51:32/51:48/51:51 | T/A |
| HG00671 | 15:02 | 48:03:01 | T/A |
| HG00701 | 15:02 | 46:01:01/46:15N | T/A |
| NA18559 | 15:02 | 37:01:01 | T/A |
| NA18639 | 15:02 | 54:01/54:17 | T/A |
| NA18630 | 15:02 | 40:01:01/40:01:02/40:55 | T/A |
| NA18152 | 15:02 | 40:01:01/40:01:02/40:55 | T/A |
| NA18674 | 15:02 | 40:01:01/40:01:02/40:55 | T/A |
| NA17999 | 15:02 | 40:01:01/40:01:02/40:55 | T/A |
| NA18127 | 15:02 | 27:04:01 | T/A |
| NA17988 | 15:02 | 40:01:01/40:01:02/40:55 | T/A |
| NA17997 | 15:02 | 38:02:01/38:18 | T/A |
| NA18118 | 15:02 | 13:01:01 | T/A |
| NA18122 | 15:02 | 15:02 | T/T |
| NA17982 | 15:02 | 35:05 | T/A |
| NA17977 | 15:02 | 55:02:01 | T/A |
| NA17969 | 15:02 | 37:01:01 | T/A |
| IHW09182 | 15:02 | 18:01 | T/A |
| IHW09185 | 15:02 | 46:01 | T/A |
| IHW09186 | 15:02 | 46:01 | T/A |
| IHW09189 | 15:02 | 46:01 | T/A |
| IHW09199 | 15:02 | 15:13 | T/T |
| IHW09237 | 15:02 | 55:02BCTX | T/A |
| IHW09432 | 15:02 | 38:02UF | T/A |
